# Numerical investigation of liquid-Cooled battery thermal management system configurations for a lithium-ion battery pack with experimental validation

**DOI:** 10.1038/s41598-026-62846-z

**Published:** 2026-07-23

**Authors:** Abdelrahman O. Ali, Osama Abdelrehim, Mahmoud M. Saafan, Mohamed R. Elmarghany, Ahmed M. Hamed

**Affiliations:** 1 Mechanical Power Engineering Department, Faculty of Engineering, Mansoura University, Mansoura, Egypt; 2 Computers and Control Systems Engineering Department, Faculty of Engineering, Mansoura University, Mansoura, Egypt

**Keywords:** Battery Thermal Management System (BTMS), Liquid Cooling, Lithium-Ion Battery, Conjugate Heat Transfer, Sony VTC6, Energy science and technology, Engineering

## Abstract

Efficient thermal management is crucial for lithium-ion battery safety and longevity in Electric Vehicles (EVs). This study presents a numerical investigation and experimental validation of liquid cooling strategies for NMC lithium-ion battery modules, comparing serpentine (Configuration 1), parallel (Configuration 2), and parallel/counter-flow hybrid (Configuration 3) layouts. A custom-built 4-cell test rig validated against CFD simulations showed close agreement, with relative deviations below 0.6%, confirming model reliability. Under regulated flow conditions, clear differences in thermal and hydraulic behavior were observed. Configuration 1 exhibited the highest thermal stress, with peak temperatures reaching 75.7 $\:^\circ\:C$ (cell 7) and 74.1$\:\:^\circ\:C$ (cell 18) due to downstream heat accumulation and limited contact area, alongside the largest temperature difference (ΔT = 11.0 K) and highest pressure drop (27.3 Pa). Configuration 2 reduced peak pack temperature to 70.6 $\:^\circ\:C$ at cell 18 (6.7% lower vs. Configuration 1) and 64.7 $\:^\circ\:C$ at cell 7 (14.8% lower), achieving the best intra-cell uniformity (σ = 0.47 K, CV = 5.6%) and lowest pressure drop (9.1 Pa). Configuration 3 offered the most balanced cooling, lowering maximum cell temperature by 7.6% compared to Configuration 1 and 0.9% compared to Configuration 2, while the maximum temperature of cell 18 decreased by 11.8%. It achieved the narrowest inter-cell ΔT (4.1 K), a 62.5% and 11% reduction versus Configurations 1 and 2, respectively, with the lowest σ = 1.0 K (CV = 0.32%). Overall, serpentine cooling is simple but thermally inefficient, parallel flow is the most energy-efficient, and the hybrid parallel/counter-flow design delivers the best overall thermal balance while lowering the required pumping power by about 89%, making it the most suitable layout for safe and reliable EV battery operation.

## Introduction

Rapid electrification in the automotive sector and consumer electronics has increased the demand for high-performance lithium-ion (Li-ion) batteries. Thermal stability is crucial for safety, efficiency, and lifespan^1^. High C-rates, ambient temperature fluctuations, and prolonged cycling impose high thermal loads^2^, where the C-rate denotes the charge or discharge current relative to the battery’s nominal capacity. Inadequate thermal management causes non-uniform temperature distribution, which accelerates aging, degrades performance, and can trigger thermal runaway^3^. High temperatures accelerate electrolyte decomposition and electrode degradation^4^. Low temperatures reduce ion mobility and depress power output^5^. Because these electrical, thermal, and aging mechanisms are strongly coupled, recent work stresses joint electro-thermal–aging state estimation to capture their interaction in operating packs^6^, while exergy-based analyses that explicitly account for lithium plating quantify the efficiency and safety penalties incurred when cells operate outside this window^7^. Thus, effective thermal management ensures that batteries operate within an optimal temperature range, typically between 15 $\:^\circ\:C$ and 40 $\:^\circ\:C$^8,9^.

**Fig. 1:**
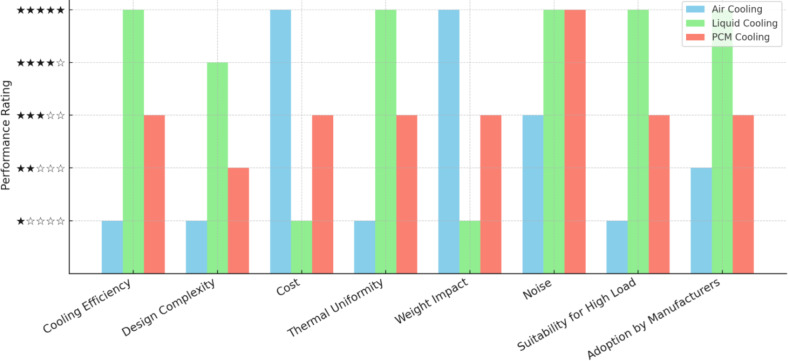
Battery Cooling Strategies in EVs Comparison^19^.

Battery thermal management strategies fall into passive and active cooling techniques. Passive methods include Phase Change Materials (PCMs) and natural convection, offering simplicity but provide limited thermal performance^10^. Active cooling, including air and liquid systems, provides higher heat dissipation and better temperature uniformity, though it requires auxiliary power and added controls^11,12^. Figure 1 compares different cooling strategies on cooling effectiveness, design complexity, cost, thermal uniformity, weight impact, noise, high loads performance, and manufacturer adoption. Liquid cooling stands out for stable performance under high-load and fast-charging conditions and for compatibility with compact battery pack designs. It balances performance and scalability, making it adopted by many modern Electric Vehicle (EV) platforms. This comparison helps select thermal solutions tailored to specific EV design and operational requirements. Comprehensive reviews of energy-efficient liquid cooling for high-energy-density Li-ion batteries reinforce this direction, consolidating both heat-extraction and thermal-runaway-suppression strategies across cell formats^13^. At the vehicle level, system-level modelling further situates the thermal subsystem within the broader energy architecture, including emerging dual-energy-storage powertrains^14^.

Liquid cooling, which is classified into direct and indirect cooling techniques, delivers high thermal performance due to the high heat capacity and density of different used coolants. Direct cooling, such as immersion cooling submerges cells in a dielectric (non-conductive) fluid, enabling direct contact convection and low thermal interface resistance^15^. It reduces temperature gradients, suppress hotspots, and can improve performance and lifespan^16^. A Channeled Dielectric Fluid Immersion Cooling (C-DFIC) system for high-energy Lithium Titanate Oxide (LTO) cells is presented in^17^, reduced maximum temperature by 17.8% and improved temperature uniformity by 33.3% versus a conventional design. With HFE-6120 at 0.5 L/min, aged cell temperature stayed below 26.1$\:\:^\circ\:C$ (299.2 $\:K$) after 3972 US06 drive cycle repeats. However, immersion cooling also presents several challenges, such as system complexity, leak-tight enclosure, added fluid weight, and fluid-material compatibility. High fluid cost and maintenance still limit adoption in EVs^18^.

Indirect liquid cooling, using cold plates with serpentine channels, is widely used in Li-ion EV packs because it balances cooling performance, design flexibility, and safety. Coolant circulates through channels adjacent to cells, removing heat without electrical contact^20^. Key advantages include high heat removal, compatibility with modular packs, and tighter temperature uniformity than air or PCM cooling^21^. However, it adds system complexity, weight, cost, and seal maintenance. Despite these trade-offs, EV manufacturers favor it for reliability, scalability, and robust control under high load and fast charge conditions^22^. Recent numerical channel-design studies continue to refine coolant distribution and temperature uniformity in cold plates; a diamond-shaped flow-channel architecture, for instance, has been proposed to homogenize flow and lower cell temperature gradients^23^. Complementing geometry-focused work, coupled loss and finite-element thermal models of production fast-charging modules resolve heat generation and temperature fields under aggressive charging, linking cell-level losses to module-scale thermal response^24^. Beyond physics-based Computational Fluid Dynamics (CFD) and Finite Element Analysis (FEA), data-driven and digital methods are increasingly deployed for real-time thermal awareness: optimized random-forest models estimate cell surface temperature for closed-loop BTMS control^25^, and digital-twin frameworks fuse sensing with simulation to track battery-system states across their life^26^.

Indirect liquid cooling employs several architectures including cooling tubes, jackets, helical coils, serpentine channels, and hybrid designs. Table 1 summarizes recent studies on channel design for cylindrical Li-ion cells, covering Configurations, techniques, and thermal performance from experiments and simulations^27–40^–^41^. Vertical tube layouts (e.g., for Li-ion iron phosphate) improve temperature uniformity and thermal stability versus horizontal arrangements but raise mechanical complexity and packing constraints. Cooling jackets deliver strong uniformity with multi-directional flow^35^. Serial/parallel arrangements also affect performance. Parallel Configurations lower thermal resistance and suppress hotspots^31^. Innovative designs such as bionic hexagonal channels, double helical coils, and external PCM composites further reduce temperature gradients^40^. Serpentine channels deliver cooling performance comparable to U-shaped layouts but with lower pressure drop, improving thermal-hydraulic tradeoffs in EV modules^28,33^. Counterflow routing, where adjacent paths carry coolant in opposite directions, further reduces through module temperature gradients. Hybrid designs that combine parallel and counter-flow remain understudied in 6s4p-type packs, representing a gap this study addresses.

Despite these advances, the studies summarized in Table 1 share three limitations that constrain their relevance to real EV modules: (i) they predominantly examine simplified cell arrays or single-layer modules rather than electrically realistic series–parallel packs; (ii) most validate cooling only up to moderate discharge rates (≤ 2 C), leaving high-load behavior unverified; and (iii) they typically optimize a single channel geometry rather than benchmarking competing topologies under an identical flow budget. Hybrid layouts combining parallel and counter-flow routing, in particular, remain largely unexplored for compact, high-power Configurations such as 6s4p cylindrical packs.

**Table 1:** Indirect liquid cooling designs and Configurations.

S.*N*.	Cooling System Type	Type of Battery	Cooling Technique Used	Design of BTMS (Cooling Plate/Channel Geometry)	Type of Study	Significant Remarks	Refs.
1	Indirect Liquid Cooling	18,650 Cylindrical Li-ion	Single-phase forced convection (Water/Glycol)	Stepped-channel liquid-cooled BTMS. Investigated channel width, cell-to-cell lateral spacing, contact height, and angle.	Numerical Simulation	Optimized design significantly reduced weight (54.08%) while maintaining acceptable temperature control (T_max_ increase by only 2.52 $\:K$). Channel width was identified as the most critical parameter.	^27^
2	Indirect Liquid Cooling	18,650 Cylindrical Li-ion	Single-phase forced convection (Water/Glycol)	Converging-diverging liquid cooling channel with wave-shaped structure (based on serpentine/U-shaped). Four geometrical Configurations analyzed.	Numerical Simulation	Optimized design reduced peak internal temperature by 20.6% compared to other designs. Identified importance of Reynolds number, arc depth, and cell spacing. Optimal for 2 C discharge.	^28^
3	Indirect Liquid Cooling	18,650 Cylindrical Li-ion	Single-phase forced convection (Water, Nanofluids)	Mini-channel fins heat sink units are integrated into cooling channels. Four models with different fin Configurations (e.g., Model II with lowest max temp).	Numerical Analysis & Experimental Testing	Demonstrated significant thermal cooling enhancement. For Model II, Tmax was 30.10$\:\:^\circ\:C$ and temperature gradient was 1.48$\:\:^\circ\:C$, exploring various heat transfer enhancement approaches.	^34^
4	Indirect Liquid Cooling	18,650 Cylindrical Li-ion	Single-phase forced convection	Gradient contact surface angle in cooling jacket for 18,650 cells.	Numerical Simulation	Achieved good cooling performance with a temperature gradient of 2.58 $\:^\circ\:C$ at 2 C discharge for a 15° contact surface angle.	^35^
5	Indirect Liquid Cooling	18,650 Cylindrical Li-ion	Single-phase forced convection	Liquid-cooled cold plate with Gradually Varied Circular Notched Fins (GV-CNF).	Numerical Simulation (CFD)	GV-CNF design showed 41.1% improvement in comprehensive performance (temperature uniformity, max temp, pressure drop) over traditional circular fins. Optimal channel height identified.	^36^
6	Indirect Liquid Cooling	18,650 Cylindrical Li-ion	Single-phase forced convection	Optimizing two inlets/outputs, channel widths, and flow orientations in a liquid cooling BTMS.	Numerical Simulation	This type of study focuses on flow distribution and channel Configurations to improve overall cooling, particularly for 18,650 packs. Results show improved temperature uniformity and reduce maximum temperatures by adjusting these parameters.	^37^
7	Indirect Liquid Cooling	18,650 Cylindrical Li-ion	Single-phase forced convection	Serpentine channel with redesigned baffles on the inner surface.	Numerical Simulation	Significant improvement in temperature uniformity and heat transfer between cells and coolant due to enhanced mixing flow in the channel.	^38^
8	Indirect Liquid Cooling	18,650 Cylindrical Li-ion	Single-phase forced convection	Wavy cooling channel liquid-cooled system.	Numerical Simulation	At 5 C discharge, this design reduced the highest temperature by 12.80 $\:^\circ\:C$ and temperature gradient by 5.30 $\:^\circ\:C$.	^39^
9	Indirect Liquid Cooling	18,650 Cylindrical Li-ion	Single-phase forced convection	Helical vs. Linear channel designs in a cold plate.	Numerical Simulation (CFD)	Linear Channel Design (LCD) proved superior in both maximum temperature reduction and thermal uniformity (1.796 $\:K$ lower Tmax, 8.740 $\:K$ lower ΔT) compared to Helical Channel Design (HCD).	^40^
10	Indirect Liquid Cooling	18,650 Cylindrical Li-ion	Single-phase forced convection	Liquid-cooled plate with variable density topology optimization for channel design.	Numerical Simulation	Reduced maximum battery module temperature by 2.4% (from 27.88$\:\:^\circ C$ to 27.21 $\:^\circ C$) and temperature difference by 13.2% (from 5.7 $\:^\circ C$ to 4.95 $\:^\circ C$), meeting performance requirements.	^30^
11	Indirect Liquid Cooling	18,650 Cylindrical Li-ion	Single-phase forced convection (Water)	Microchannel-embedded cylinder cooling system.	Numerical Simulation	Optimized design kept the maximum temperature at 27.80 $\:^\circ\:C$ with a temperature gradient of 0.80 $\:^\circ\:C$ for discharge rates of 1 C, 1.5 C, and 2 C.	^41^
12	Direct Liquid Cooling (Immersion)	18,650 Cylindrical Li-ion	Dielectric fluid immersion	No complex channels; cells directly immersed in SF33-based liquid.	Numerical Simulation & Experimental Validation	Effectively mitigates heat accumulation and provides notable advantages in temperature control and equalization, with greatly reduced cooling energy consumption compared to forced air cooling.	^29^
13	Liquid	18,650 cylindrical	Serial & parallel tubing	Serial-parallel channels	Simulation + Experimental	Parallel system outperforms serial; glycol solution better than water	^31^
14	Liquid	18,650-type cylindrical	Wavy multi-channel tubes	Multi-channel embedded pipes	Simulation + Experimental	Wavy design increases heat exchange even at high discharge	^32^
15	Liquid	18,650-type cylindrical	Serpentine/U-shaped channels	Flow optimization channels	Simulation	U-shaped channels reduce pressure loss; same thermal effect as serpentine	^33^ 33
16	Indirect Liquid Cooling	18,650 (Sony VTC6), 6s4p pack	Single-phase forced convection (water)	Serpentine vs. parallel vs. hybrid parallel–counter-flow, normalized flow	Numerical Simulation & Experimental Validation	Hybrid gives narrowest ΔT (4.1 K); 62.5%/11% lower than serpentine/parallel; validated at 3 C	Present study

The present work addresses these gaps by experimentally validating a CFD model against a custom 4-cell rig (steady-state error within ± 1.6 K, relative deviation below 0.6%) and then benchmarking three topologies — single serpentine (Config. 1), three parallel channels (Config. 2), and a hybrid two-parallel/one-counter-flow design (Config. 3) — in a realistic 6s4p Sony VTC6 pack under a normalized total flow rate and discharge rates up to 5 C. The main contributions are: (1) a like-for-like, flow-normalized comparison of serpentine, parallel, and hybrid counter-flow cooling at module level, in which maximum cell temperature, inter- and intra-cell uniformity, and channel pressure drop are all reported; (2) experimental validation of both the numerical model and the serpentine-manufactured cooling channel, confirming that the fabricated geometry reproduces the idealized design intent; (3) quantitative evaluation of the hybrid design’s thermal and hydraulic advantage under high load (up to 5 C), where it attains the narrowest inter-cell temperature difference (ΔT = 4.1 K — 62.5% and 11% lower than the serpentine and parallel layouts), while the parallel and hybrid layouts cut pumping power by ~ 89% at matched flow, giving a thermal-hydraulic efficiency nearly an order of magnitude higher; and (4) a practical design recommendation showing that the parallel layout already captures most of the benefit at lower complexity, with diminishing returns beyond moderate flow rates.

The remainder of the paper is as follows: Sect. 2 describes the system design and simulation setup; Sect. 3 outlines the governing equations and modeling approach; Sect. 4 details the simulation tools and experimental validation procedures; Sect. 5 discusses the simulation results; Sect. 6 concludes the paper with key findings and future research directions.

## Battery thermal management design

**Fig. 2:**
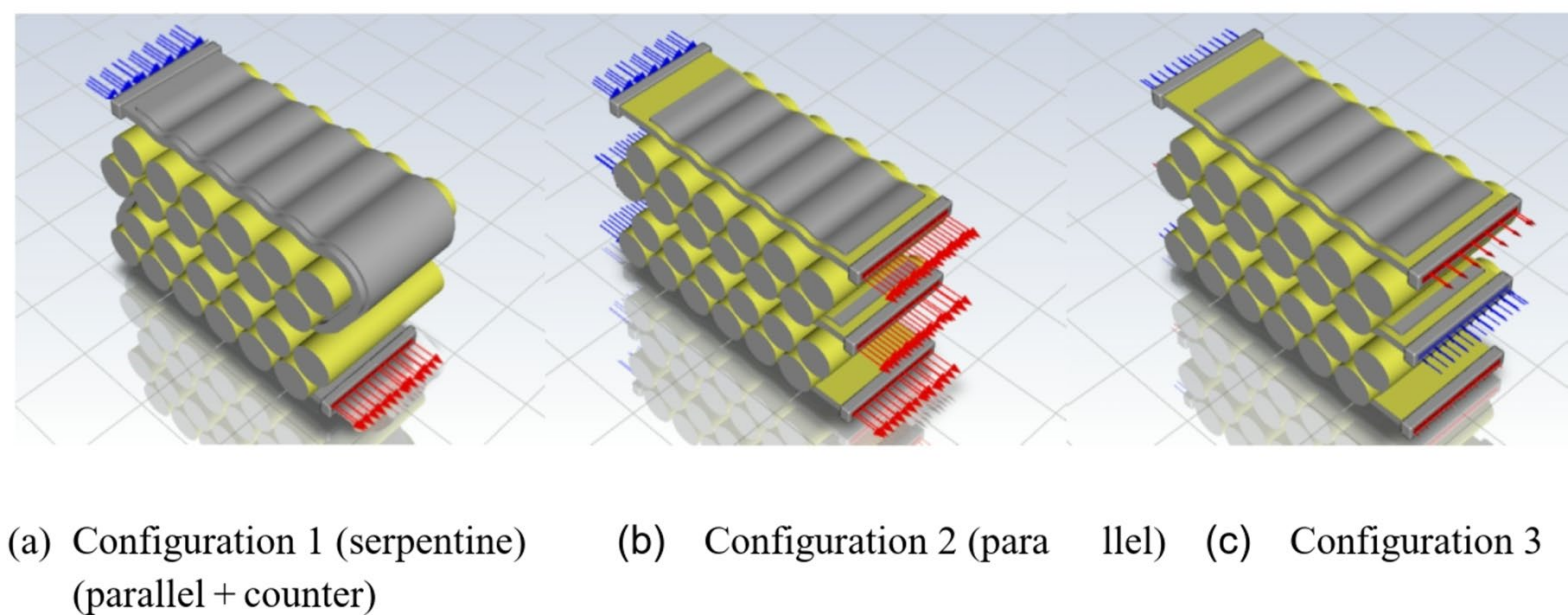
Schematic illustration of three liquid cooling channel Configurations for cylindrical Li-ion battery packs (a) Configuration 1, (b) Configuration 2, (c) Configuration 3.

**Fig. 3:**
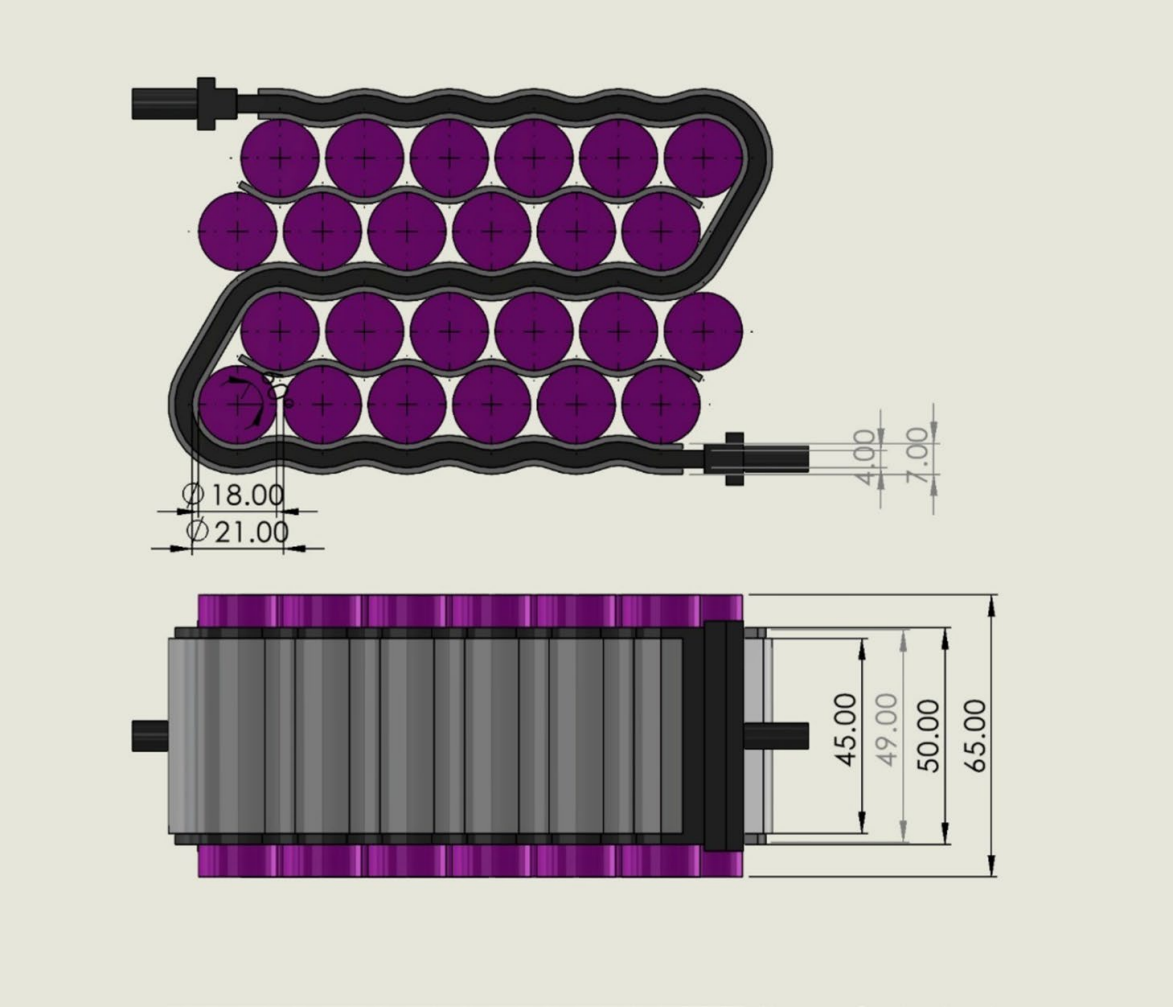
Dimensions of the cylindrical Li-ion battery pack.

The system models three battery thermal management Configurations (serpentine, parallel, and a hybrid counter-flow) within a realistic 6s4p battery pack. Figure 2 illustrates the liquid cooling channel layout. In Configuration 1 (Fig. 2a), a single serpentine tube traverses the entire battery pack, directing coolant sequentially across all 24 cells. The serpentine tube layout follows the approach described in^42^, originally used in Tesla battery systems^43^. Configuration 2 (Fig. 2b) employs three parallel tubes placed between cell rows; the flow splits and runs simultaneously in each branch. Configuration 3 (Fig. 2c) employs two parallel tubes with one counter-flow tube to create bidirectional routing. All cooling channels are made of aluminum with 0.5 $\:mm$ wall thickness and an internal cross section of 50 $\:mm$ × 4 $\:mm$ as shown in Fig. 3. This compact geometry maintains stiffness and limits system weight while providing adequate coolant flow area. A 1.5 $\:mm$ Thermal Interface Material (TIM) layer is placed between each cooling tube and the cylindrical cell surfaces to enhance the contact and reduce interfacial resistance.

This modular aluminum-tube-based approach ensures scalable integration into EV packs with minimal added weight and high structural compatibility with existing 18,650 cylindrical cell arrays. Its low-profile height and selective thickness ensure efficient space usage, a critical consideration for EV architecture. The design’s feasibility is further supported by Computational Fluid Dynamics (CFD) simulations validating its performance across multiple flow and heat generation scenarios.

The thermal management system used a 6s4p Li-ion pack built from Sony VTC6 cells which are widely used in high-performance applications due to their high capacity and good discharge characteristics. The Sony VTC6 is a high-drain 18,650 cell employing a nickel–manganese–cobalt oxide (NMC) cathode, a graphite anode, and a lithium-salt (LiPF₆) organic carbonate electrolyte. The complete cell- and pack-level technical specifications are summarized in Table 2. The 24 cells are assembled in a 6-series × 4-parallel (6s4p) configuration, providing a nominal pack voltage of 21.6 V and a capacity of 12 Ah.

**Table 2:** Technical specifications of the Sony VTC6 18,650 cell and the assembled 6s4p pack.

Parameter	Specification
Cell chemistry	
Cathode material	Nickel–manganese–cobalt oxide (NMC)
Anode material	Graphite
Electrolyte	LiPF₆-based organic carbonate
Cell electrical ratings	
Nominal voltage	3.6 V
Rated capacity	3000 mAh
Max. continuous discharge current	15 A
Nominal energy	≈ 10.8 Wh
Cell physical properties	
Diameter	18.4 mm
Length	65 mm
Mass	46.6 g
Pack-level ratings (6s4p, 24 cells)	
Configuration	6 series × 4 parallel
Nominal voltage	21.6 V
Capacity	12 Ah

### Material properties

In the thermal simulation of the battery cooling system, each material plays a critical role in determining heat transfer efficiency and overall thermal stability. The Sony VTC6 Li-ion cell is modeled as a low thermal conductivity solid, emphasizing the importance of effective heat extraction. TIM, placed between the battery surface and cooling components, reduces contact resistance, and improves heat spreading between cells and the cooling channels. The channels are made of aluminum — chosen for its high conductivity, low density, and manufacturability — providing a rapid path from TIM to the coolant. Water serves as the coolant because of its high specific heat capacity, which allows it to absorb significant amounts of heat with minimal temperature rise. Together, these materials create a continuous thermal pathway from the cell core to the heat sink. Table 3 summarizes the thermal properties, thermal conductivity, specific heat, and density, of each material used in the model.

**Table 3:** Thermal properties of materials used in the battery cooling system.

Material	Thermal Conductivity $\:(\boldsymbol{W}/\boldsymbol{m} \cdot\boldsymbol{K})$	Specific Heat $\:(\boldsymbol{J}/\boldsymbol{k}\boldsymbol{g} \cdot\boldsymbol{K})$	Density $\:(\boldsymbol{k}\boldsymbol{g}/\boldsymbol{m}^3)$
Sony VTC6 Battery Cell^44^^[,45^	3.0	880	2871.8
Thermal Interface Material (TIM)^46^	2.0	1000	2500
Aluminum (Cooling Channel)^47^	202.4	871	2719
Water (Coolant)^47^	0.6	4182	998.2

## Governing equations and thermal modeling approach

The governing equations and thermal modeling approach presented in this section provide a foundational framework for simulating heat generation, transfer, and dissipation within the battery pack, enabling accurate prediction of thermal behavior under various operating conditions.

### Fluid flow equations

The coolant flow inside the tubes was assumed laminar, given the low Reynolds numbers calculated for the flow rates considered. Thus, the steady, incompressible continuity and momentum equations govern the flow^47,48^:

Continuity equation:

1 $$\:\nabla\:\cdot\:\mathbf{v}=0$$

Momentum conservation:

2 $$\:\rho\:\left(\mathbf{v}\cdot\:\nabla\:\mathbf{v}\right)=-\nabla\:p+\mu\:{\nabla\:}^{2}\mathbf{v}$$

Where: $\:p$, $\:\mu\:$, $\:\mathrm{v}$, and $\:\rho\:$ are the pressure ($\:Pa$), dynamic viscosity ($\:Pa.s$), velocity vector (m/s), and density (kg/m^3^).

### Energy conservation

The thermal behavior of the battery pack was modeled using the energy conservation equation, accounting for internal heat generation within the battery cells and heat conduction through solid materials and through the cooling water^47,49^:

3 $$\:\rho\:{C}_{p}\mathbf{v}\cdot\:\nabla\:T=\nabla\:\cdot\:\left(k\nabla\:T\right)+\stackrel{\prime }{q}\:\:$$

Where $\:{C}_{p}$, $\:T$, $\:k$, and $\:\stackrel{\prime }{q}$ are the specific heat ($\:J/kg.\:K$), temperature ($\:K$), thermal conductivity (W/m.K), and volumetric heat generation rate ($\:W/{m}^{3}$).

### Heat generation in battery cells

The internal heat generation rate inside each cell $\:\left(\stackrel{\prime }{q}\right)$ was calculated using a simplified Bernardi equation^50^:

4 $$\:\stackrel{\prime }{q}=\frac{1}{V}\left({I}^{2}{R}_{i}+IT\frac{d{U}_{0}}{dT}\right)$$

Where V, I, $\:{R}_{i}$, and $\:\frac{d{U}_{0}}{dT}$ are the cell volume ($\:{m}^{3}$), discharge current ($\:A$), internal resistance ($\:\varOmega\:)$, and entropic heat coefficient ($\:V/K$). For the battery used here (Sony VTC6), a typical value of $\:\frac{d{U}_{0}}{dT}$ is $\:0.01116\text{}V/K$^51^. Internal resistance values were assumed based on manufacturer data and experimental measurements of the battery, with an average value of $\:{R}_{i}=21.85\text{}m\varOmega\:$.

### Thermal uniformity metrics

Battery thermal analysis distinguishes between inter-cell thermal uniformity and intra-cell thermal uniformity. Inter-cell thermal uniformity assesses the temperature consistency among all cells in the pack and is quantified using statistical metrics such as the standard deviation and coefficient of variation of the average cell temperatures. This metric is crucial for evaluating pack-level balance, preventing uneven aging, and supporting State-of-Charge (SOC) and State-of-Health (SOH) equalization strategies in BMS. If $\:{T}_{i}$ is the temperature of the $\:{i}^{th}$ cell at a given time, then the mean temperature $\:{\bar T}\:$, standard deviation $\:{\sigma\:}_{T}\:$, and coefficient of variation (CV) can be calculated as shown in Eqs. 5–7^52^. Note that a lower $\:{\sigma\:}_{T}$ indicates better uniformity.

5 $$\:{\bar T}=\frac{1}{N}\sum\:_{i=1}^{N}\:{T}_{i}$$

6 $$\:{\sigma\:}_{T}=\sqrt{\frac{1}{N}\sum\:_{i=1}^{N}\:\:{\left({T}_{i}-{\bar T}\right)}^{2}}$$

7 $$\:\mathrm{C}\mathrm{V}=\frac{{\sigma\:}_{T}}{\bar T}\times\:100$$

On the other hand, intra-cell thermal uniformity evaluates the temperature gradient within each individual cell, typically expressed as:

8 $$\:{\Delta\:}{T}_{\mathrm{c}\mathrm{e}\mathrm{l}\mathrm{l},i}={T}_{\mathrm{m}\mathrm{a}\mathrm{x},i}-{T}_{\mathrm{m}\mathrm{i}\mathrm{n},i}\:$$

where $\:{T}_{\mathrm{m}\mathrm{a}\mathrm{x},i}$ and $\:{T}_{\mathrm{m}\mathrm{i}\mathrm{n},i}$ are the maximum and minimum temperatures observed on cell $\:i$. This metric reflects internal thermal stress that drives material degradation, electrochemical imbalances, and thermal runaway risk. Ensuring high intra-cell uniformity is especially important for safe operation and long-term cell integrity. Both types of uniformity must be analyzed together for a comprehensive evaluation of thermal performance in battery packs, especially under high C-rate or fast-charging conditions.

## Simulation tools and boundary conditions

All simulations used ANSYS Fluent 2023 R1, a commercial CFD solver. The solver was used to solve the conjugate heat transfer by coupling the solid and fluid energy equations. This setup is appropriate for battery thermal modeling.

### Solver settings

ANSYS Fluent settings were selected to capture the pack’s thermal and flow behavior. A pressure-based solver was employed due to the incompressible nature of the coolant. The simulation is conducted in a steady state mode. The flow regime was characterized using the Reynolds number based on the channel hydraulic diameter, $\:Re=\rho\:v{D}_{h}/\mu\:$, with $\:{D}_{h}=2ab/(a+b)$. For the $\:50\times\:4$ mm cross-section, $\:{D}_{h}=7.41\text{}mm$. Using water properties $\:\left(\rho\:=998.2\text{}kg/{m}^{3},\mu\:=\right.1.0\times\:{10}^{-3}\text{}Pa\cdot\:\text{}s$), the Reynolds number ranges from $\:Re\approx\:7$ at $\:0.001\text{}m/s$ to $\:Re\approx\:$ 370 at $\:0.05\text{}m/s$ ($\:\approx\:178$ at the $\:0.024\text{}m/s$ reference). These values are well below the laminar-turbulent transition ($\:\sim\:2300$), confirming that the laminar flow model adopted in the simulations is appropriate across the entire operating range. The Second-Order Upwind scheme was used for spatial discretization to enhance accuracy in capturing thermal and velocity gradients. Pressure-velocity coupling was handled using the SIMPLE algorithm. A convergence criterion of 10⁻¹⁰ was set for residuals to ensure solution stability and numerical reliability throughout the simulation.

### Boundary conditions

To accurately assess the thermal behavior of the battery pack under various operating conditions, the simulation incorporated realistic boundary conditions as outlined in Tables 4 and 5. The ambient temperature and coolant inlet temperature were both set at 25$\:\:^\circ\:C$. Uniform inlet velocities varied between 0.001 $\:m/s$ and 0.05 $\:m/s$, corresponding to flow rates ranging from 0.009 to 0.45 $\:L/min$ for Configurations 2 and 3. To maintain the same total mass flow rate in Configuration 1, the inlet velocity was set three times higher (i.e., 0.003–0.05 $\:m/s$ or 0.027–0.45 $\:L/min$). The cell heat generation rates were determined using the simplified Bernardi model^50^, incorporating both electrochemical and resistive components, and varied with discharge rates from 1 C to 5 C. A convective heat transfer coefficient of 5 $\:W/m^2\cdot K$ was used for natural heat loss to ambient air. These values ensure realistic simulation conditions that reflect typical EV battery operation under various C-rates and thermal loads. The solid and fluid domains were thermally coupled through conjugate heat transfer, allowing heat conduction in solids and convective heat transfer in fluids to be modeled simultaneously. Boundary conditions at the solid-fluid interface ensured continuity of temperature and heat flux:

9 $$\:\frac{\partial\:{T}_{s}}{\partial\:n}={k}_{f}\frac{\partial\:{T}_{f}}{\partial\:n}$$

Where subscripts $\:s$ and $\:f$ denote solid and fluid properties, respectively, and $\:n$ represents the normal direction to the interface.

**Table 4:** Heat generation at different discharge rates based on the Bernardi model.

Discharge Rate (C-rate)	1	2	3	4	5
Battery Current (A)	3	6	9	12	15
Heat Generation Rate Q. $\:(\boldsymbol{W}/\boldsymbol{m}^3)$	12,082	46,381	103,547	183,579	286,477

**Table 5:** Boundary conditions used in thermal simulation.

Parameter	Value
Ambient Temperature $\:{\boldsymbol{T}}_{\boldsymbol{a}}$	$\:{25\:}^{\circ\:}C$
Inlet Coolant Temperature $\:{\boldsymbol{T}}_{\boldsymbol{i}}$	$\:{25\:}^{\circ\:}C$
Inlet Velocity (Configurations 2 & 3)	$\:0.001-0.05\text{}m/s\:\:(0.009-0.45\text{}L/min)$
Inlet Velocity (Configuration 1)	$\:0.003-0.05\text{}m/s\:\:(0.027-0.45\text{}L/min)$
Convective Heat Transfer Coefficient of Air $\:{\boldsymbol{h}}_{\mathrm{air\:}}$	$\:5\text{}W/{m}^{2}\cdot\:\text{}K$

### Mesh generation and independence test

**Fig. 4:**
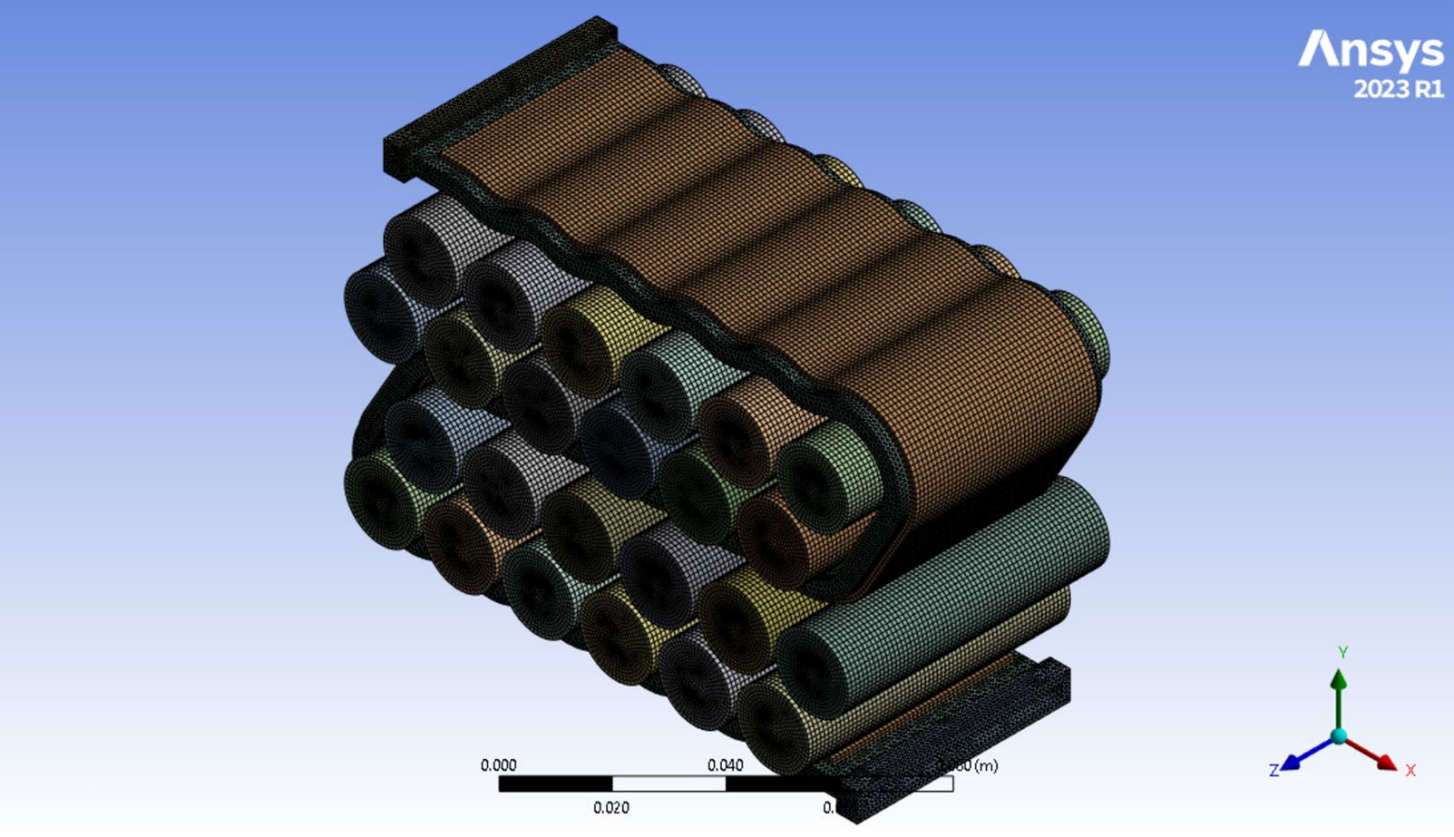
Mesh of the 6s4p cylindrical Li-ion battery module with integrated serpentine liquid-cooling channel, generated in ANSYS Meshing using structured hexahedral elements for the cells and unstructured tetrahedral elements with inflation layers near the fluid–solid interfaces.

**Fig. 5:**
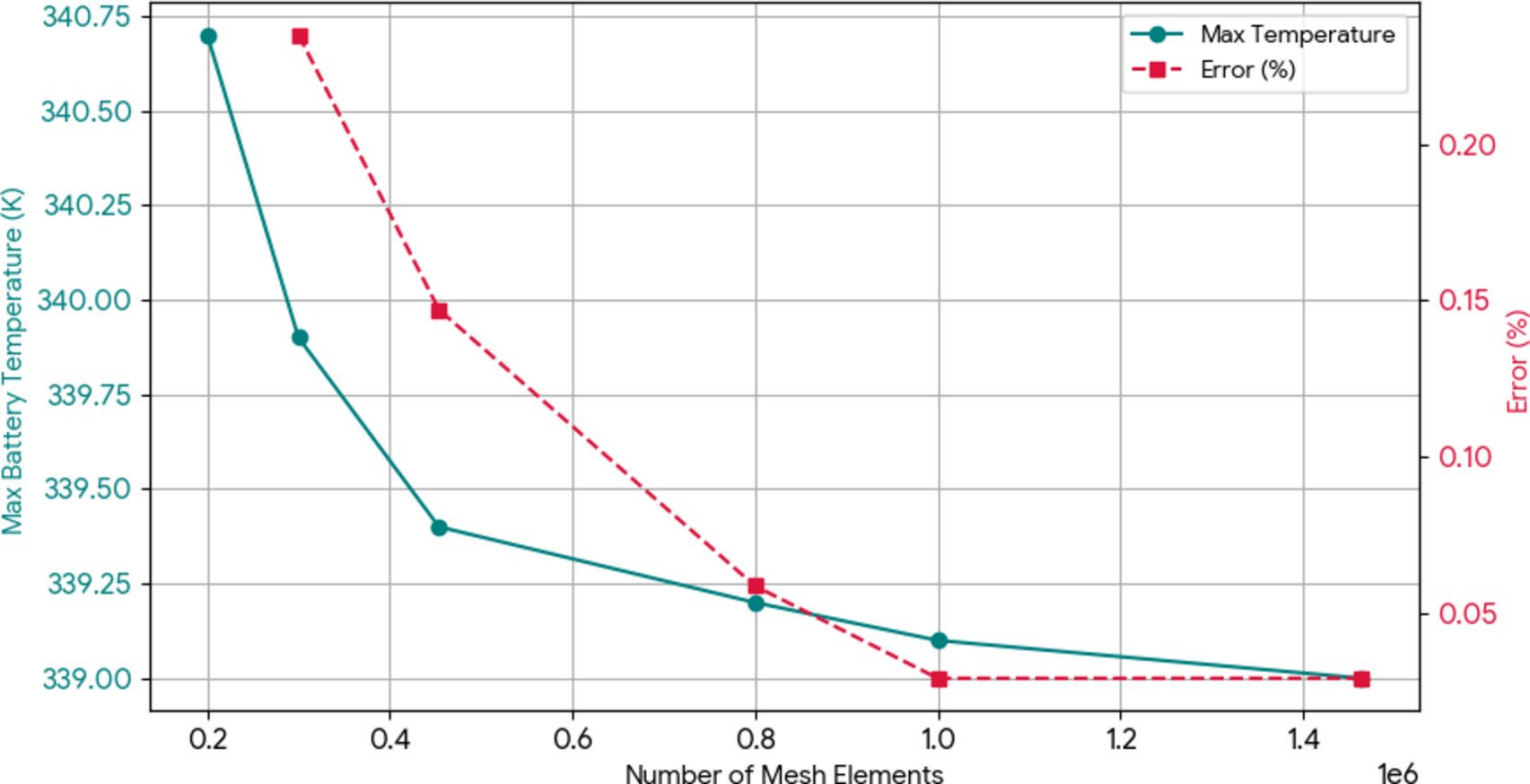
Variation of maximum battery temperature and error with different mesh element counts.

The mesh was generated in ANSYS Meshing using a combination of structured hexahedral and unstructured tetrahedral elements. A global element size of 1 $\:mm$ was applied, with local refinement around the serpentine cooling channel to capture fluid–solid interactions and accurately resolve the thermal and velocity boundary layers. Cylindrical cells were discretized with a predominantly structured hexahedral mesh to improve accuracy and reduce skewness, while the curved cooling channel and complex contact regions were meshed with unstructured tetrahedral elements for flexibility as shown in Fig. 4. The inflation-layer growth rate was 1.2. Each Configuration contained approximately 1.5 million elements. To verify mesh quality and balance computational efficiency, a mesh independence study was performed for Battery 1 in Configuration 1 under a 5 C discharge rate and a flow velocity of 0.024 $\:m/s$. As illustrated in Fig. 5, increasing the element count from 200,000 to 1,464,174 resulted in a consistent decline in the maximum battery temperature. For meshes above 1,000,000 elements, the change in cell temperature was less than 1%, with a final relative error of approximately 0.03%. Therefore, the mesh containing 1,464,174 elements with an average size of 1 mm was selected for all subsequent simulations to ensure both accuracy and computational feasibility.

### Model validation

**Fig. 6:**
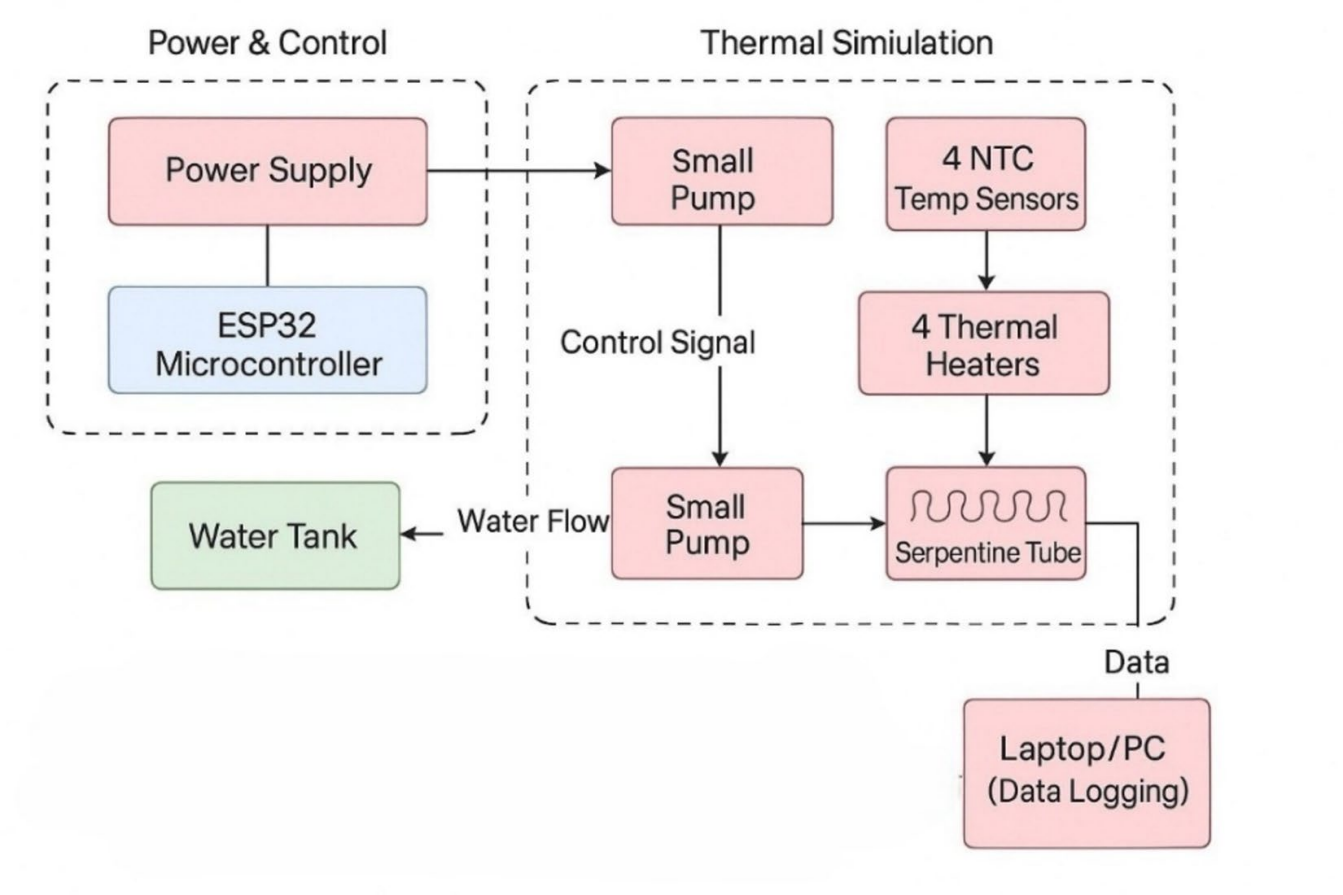
Experimental system Configuration for power delivery and control.

**Fig. 7:**
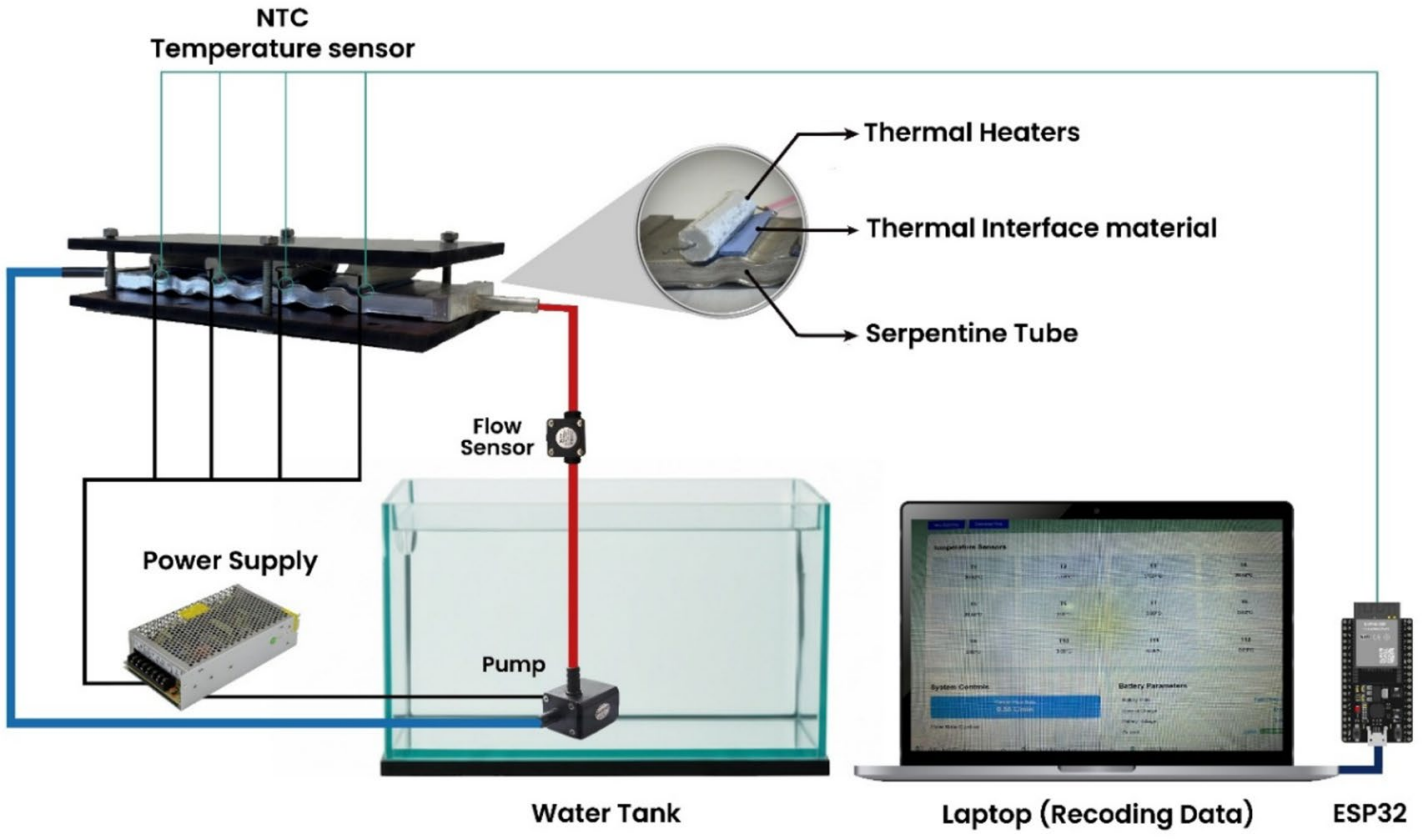
Schematic layout of key components in the experimental system.

**Fig. 8:**
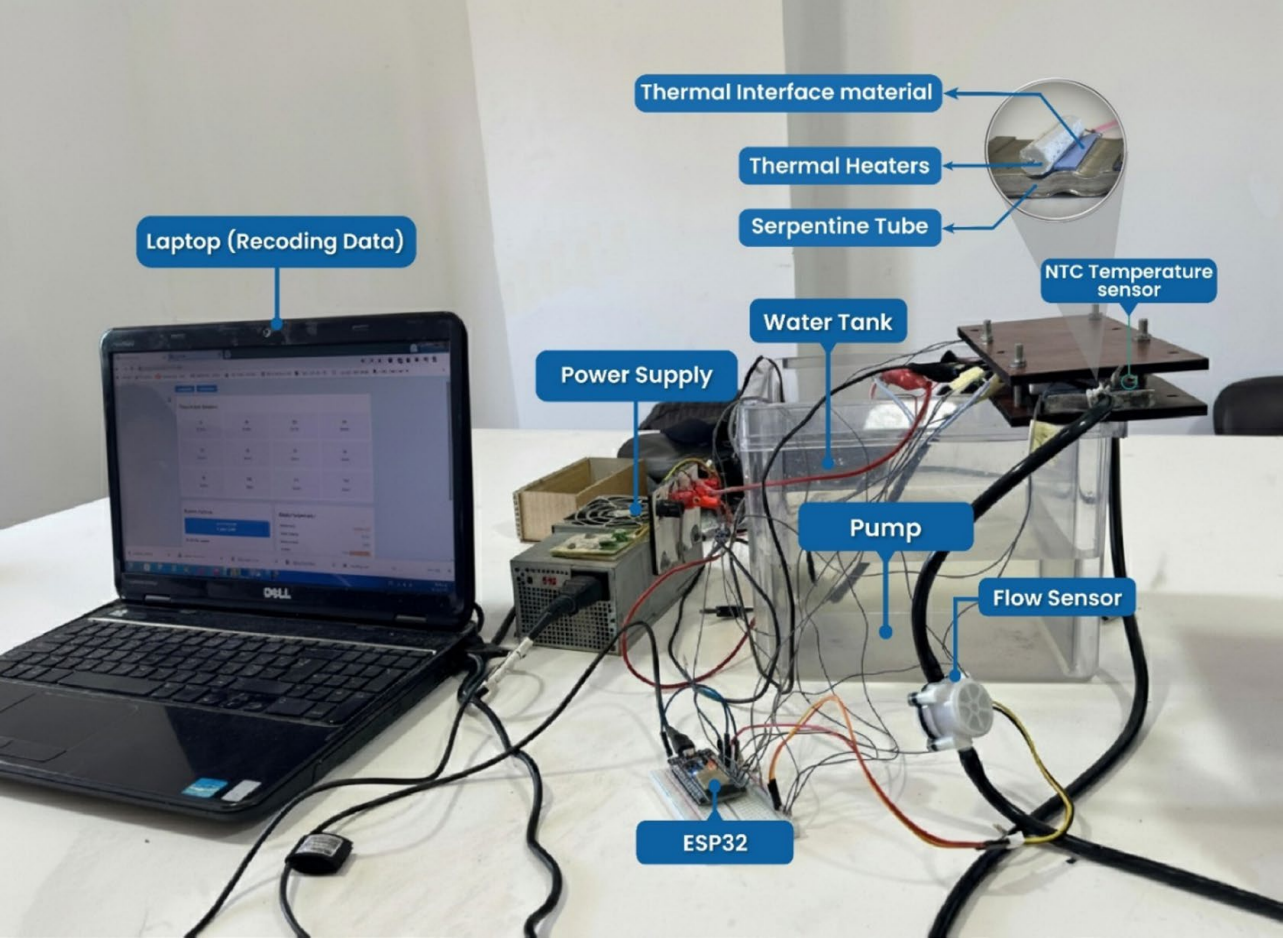
The actual experimental setup for simulating liquid cooling of Li-ion batteries using a serpentine tube (Configuration 2).

The experimental system, shown in Figs. 6, 7 and 8, was designed to simulate liquid cooling of Li-ion batteries using a serpentine flow channel. Instead of actual battery cells, four miniature ceramic thermal heaters (rated 10 $\:W$ each) were used to emulate the heat generation of 18,650-type cylindrical cells under operation; the supplied power was set so that the emulated volumetric heat load matched the 3 C cell heat-generation rate of 103,547 $\:W/m^3$ used in the simulations. Each heater is individually instrumented with a Negative Temperature Coefficient (NTC) thermistor ([10 $\:k\varOmega\:$, B = 3950 $\:K$, ± 0.2 $\:^\circ\:C$]) placed at its geometric center to measure the surface temperature. A transparent water tank (6000 $\:mL$) serves as the coolant reservoir. Water is circulated from the tank through the serpentine tube using a small DC pump (6 $\:V$, up to 2 $\:L/min$). The serpentine tube, made of aluminum with a 50 × 4 $\:mm$ internal cross-section and 0.5 $\:mm$ wall thickness, clamped between two rigid plates (Fig. 7), ensures a controlled flow path for effective heat exchange, with a 1.5 mm TIM layer at the heater–tube interface. The coolant exits the serpentine channel and returns to the water tank, establishing a closed-loop flow. A photograph of the fully assembled rig is shown in Fig. 8, providing a direct physical counterpart to the schematic in Fig. 7. An ESP32 microcontroller (ESP32-WROOM-32, 12-bit ADC) was used for system control and data acquisition as shown in Fig. 8. It regulates the coolant flow rate via a flow sensor (YF-S402, range 0.1–3.0 $\:L/min$, accuracy ± 5%) and the pump’s control signal, maintaining the target 0.27 L/min, while also recording temperature measurements from the four NTC sensors. The power supply unit provides the necessary voltage and current for both thermal heaters and the control electronics.

The schematic in Fig. 6 illustrates the system Configuration, divided into three functional blocks:

- Power & Control – comprising the power supply and ESP32 microcontroller.
- Thermal Simulation – consisting of the small pump, four thermal heaters, four NTC temperature sensors, and the serpentine tube heat exchanger.
- Instrumentation – including the water tank, flow regulation components, and a PC/laptop for real-time data logging.

This setup enables controlled investigation of coolant flow rate, temperature distribution, and heat dissipation performance in a serpentine cooling channel, providing a safe and repeatable method for evaluating liquid cooling strategies for Li-ion battery packs.

**Fig. 9:**
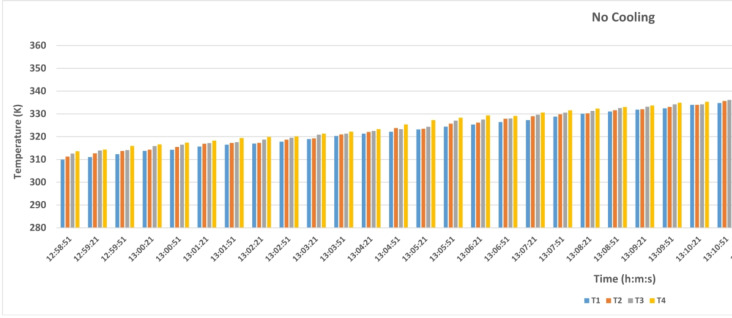
Temperature maximum distribution for experimental setup (Configuration 2) at no cooling case.

**Fig. 10:**
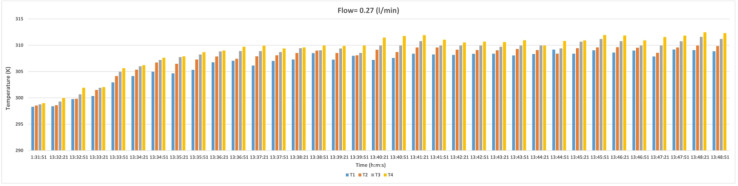
Temperature maximum distribution for experimental setup (Configuration 2) at flow = 0.27 $\:l/min$.

Under 3 C discharge conditions (I = 9 $\:A$), the thermal response of the battery pack subsection (four-cell module) was experimentally evaluated with and without serpentine liquid cooling at a flow rate of 0.27 $\:L/min$. In the absence of active cooling, as shown in Fig. 9, the maximum cell temperature reached 355.44 $\:K$ (82.3 $\:^\circ\:C$) with an average of 352.5 $\:K$ (79.3 $\:^\circ\:C$) across all sensors, clearly exceeding the safe operating threshold of 333.15 $\:K$ (60 $\:^\circ\:C$). By contrast, in Fig. 10, serpentine cooling limited the maximum temperature to 312.46 $\:K$ (39.3 $\:^\circ\:C$) and the average to 310.9 $\:K$ (37.7 $\:^\circ\:C$), corresponding to a reduction of approximately 42.98 $\:K$ (42.98 $\:^\circ\:C$, 52.2%) at the peak cell and 41.6 $\:K$ (41.6 $\:^\circ\:C$, 52.5%) in the average temperature. Furthermore, thermal non-uniformity across the cells was reduced from a 4.2 $\:K$ (4.2 $\:^\circ\:C$) spread in the uncooled case to 3.6 $\:K$ (3.6 $\:^\circ\:C$) with cooling, reflecting a 14% improvement in temperature homogeneity. Transient analysis within a 10-minute window (13:00–13:10) revealed that the heating rate decreased from 1.87 $\:K/min$ (1.87 $\:^\circ\:C/min$) without cooling to 0.85 $\:K/min$ (0.85 $\:^\circ\:C/min$) with cooling, representing a 55% reduction in thermal accumulation. These findings demonstrate that serpentine cooling not only prevents excessive temperature rise but also enhances spatial uniformity and mitigates thermal stress, thereby supporting safer operation and improved cycle life of Li-ion cells under high discharge rates.

The ANSYS thermal model was validated against a 4-cell experimental rig using the same serpentine cooling geometry. The CFD model was validated against experimental measurements obtained under a 3 C discharge rate (I = 9 $\:A$), with heat generation and coolant conditions set identical to the simulation case (Q. = 103547 $\:W/m^3$, velocity = 0.03 $\:m/s$, flow rate = 0.27 $\:L/min$). Temperature evolution was recorded at four sensor positions (T1–T4) over 35 time steps, and the steady-state temperature was defined as the average of the final five readings for each sensor.

Steady-state temperatures (average of the last five experimental readings) agree well with the CFD predictions: the absolute errors range from − 1.57 $\:K$ (− 1.57 $\:^\circ\:C$) to + 1.54 $\:K$ (+ 1.54 $\:^\circ\:C$), corresponding to relative errors of −0.51% to + 0.50%. These differences are small and lie within accepted thermal-validation tolerances (≤ 5% and ≤ 2–5 $\:K$ or ≤ 2–5 $\:^\circ\:C$ for battery thermal studies). Residuals are attributed primarily to minor flow maldistribution and local contact-resistance differences; after accounting for these effects, the CFD model reliably predicts local cell temperatures for the studied geometry and operating point.

The 4-cell sub-module was selected as the validation unit because it isolates the governing physics common to the full 6s4p pack while remaining experimentally manageable. The repeating thermal-hydraulic unit of the pack — coolant flow through the serpentine channel, conjugate heat transfer across the channel wall, boundary-layer development, and the contact resistance introduced by TIM — is fully represented at the 4-cell scale. Capturing these localized fluid–solid interactions accurately is the prerequisite for trustworthy pack-level prediction, since pack behavior is an aggregation of these same unit-cell phenomena rather than a fundamentally different regime. A further purpose of the rig was to validate the serpentine-manufactured channel against the idealized CFD geometry: machining and assembly tolerances in the serpentine tube and clamping plates can introduce flow maldistribution and contact-resistance variations not present in the CAD model, and agreement between measured and predicted temperatures confirms that the manufactured channel reproduces the intended thermal-hydraulic behavior. The close steady-state agreement (relative errors of − 0.51% to + 0.50%) therefore confirms both the fidelity of the numerical model and the manufacturing quality of the cooling channel. Therefore, the subsection CFD model is validated and can be used with confidence for local thermal performance assessment.

Table 6 provides a numerical comparison of these experimental steady-state values with the ANSYS-predicted maximum cell temperatures, showing accepted agreement confirming the reliability of the local temperature predictions. Additional full time-series goodness-of-fit analysis yielded RMSE values of [4.89, 4.17, 3.58, 3.61] K ([4.89, 4.17, 3.58, 3.61] $\:^\circ\:C$) and MAE values of [3.83, 2.78, 2.01, 2.22] K ([3.83, 2.78, 2.01, 2.22] $\:^\circ\:C$) for T1–T4, respectively. The larger RMSE/MAE values result from transient discrepancies during the warm-up phase, since ANSYS provides a single steady-state maximum value while the experimental data captures instantaneous temperature fluctuations. Overall, the close steady-state match demonstrates that the CFD framework can accurately reproduce the thermal response of the battery module under realistic operating conditions.

Following the root-sum-square method of Moffat, the measurement uncertainties were quantified by combining systematic and random components as $\:U=\sqrt{{B}^{2}+{P}^{2}}$. The NTC thermistors have a systematic accuracy of $\:\pm\:{0.2}^{\circ\:}\mathrm{C}$, and the random scatter of the steady-state readings contributed below $\:\pm\:0.2\text{}K$, giving a combined temperature-measurement uncertainty of approximately $\:\pm\:0.27\text{}K$ per sensor. The YF-S402 flow sensor is accurate to $\:\pm\:5\mathrm{\%}$ of reading ($\:\pm\:0.0135\text{}L/min$ at 0.27 $\:L/min$), while the 12-bit data-acquisition resolution ($\:<0.1\text{}\mathrm{K}$) is negligible. Since the maximum numerical-experimental deviation ($\:1.57\text{}K,<0.6\mathrm{\%}$) exceeds this measurement uncertainty, it is attributed to minor flow maldistribution and contact-resistance effects rather than sensor error, confirming that the CFD model reliably reproduces the measured thermal response.

The present validation was conducted at a single representative operating point (3 C, 0.27 L/min). As the validated physics are governed by the unit-cell interactions described above rather than by pack size, the model is considered reliable for scaling to the 24-cell domain; nevertheless, full-scale 24-cell experimental validation under a range of flow rates and discharge conditions is identified as a valuable extension and is planned for future work.

**Table 6:** Numerical experimental comparison (steady-state).

Sensor	Numerical T_max_ (K)	Exp steady mean (average of the last five experimental readings) (K)	Relative error (%)
T1	310.38	308.81	0.51%
T2	310.25	309.50	0.24%
T3	309.84	310.69	0.27%
T4	310.27	311.82	0.50%

## Results and discussion

This section compares the thermal performance across the three cooling Configurations: serpentine, parallel and counter-flow. In EV applications, Li-ion packs face a wide C-rate range from daily driving ($\:\approx\:$ 0.2–0.5 C) to short high-power transients during rapid acceleration or regenerative braking, which impose high C-rates that can reach 5 C. Although these events last only seconds, so pack average temperature rises slowly, local heating can be severe and can govern BTMS design. In the simulation, discharge rates from 1 C to 5 C are tested. Although 5 C exceeds typical sustained operation, it probes performance limits and robustness under worst-case thermal loading. These conditions reveal thermal gradients, hotspot formation, and the effects of surface contact and flow distribution. The 5 C cases validate the cooling capacity and structural resilience and inform optimization, supporting safer, longer-lasting EV packs.

### Temperature distribution

**Fig. 11:**
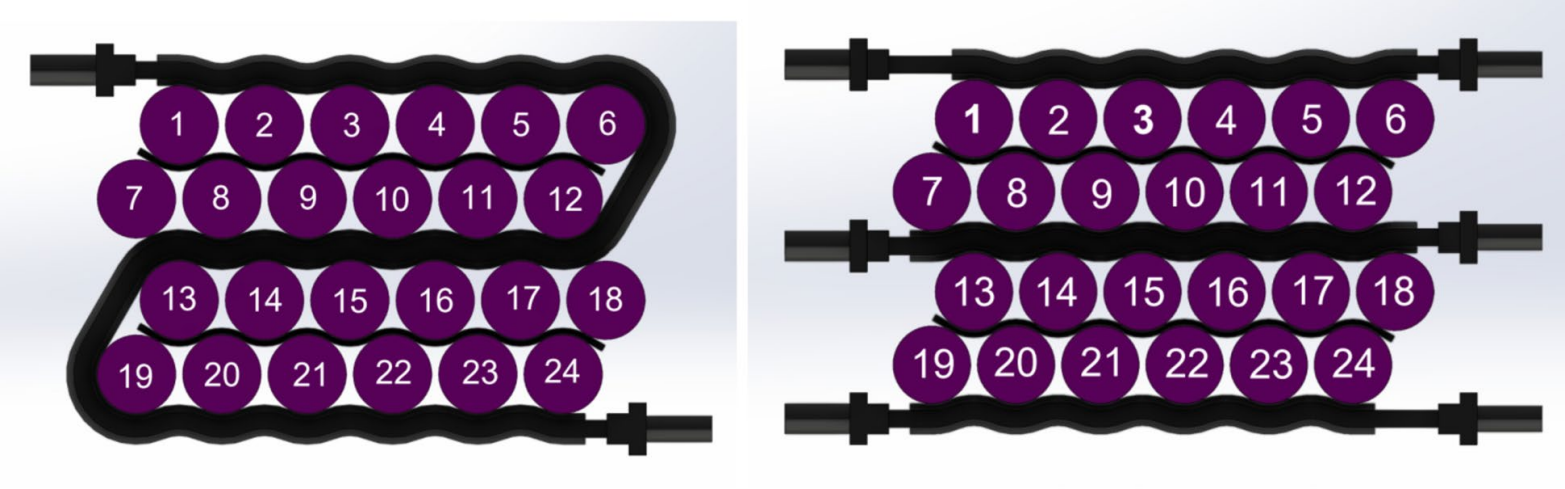
(**a**) Cell numbering scheme for Configuration 1& (**b**) Cell numbering layout for Configurations 2 and 3,.

Temperature contours were analyzed for each Configuration. The cell numbering across the three Configurations follows a consistent top-down, left to right order: row 1, cells 1 to 6; row 2, cells 7 to 12; row 3, cells 13 to 18; row 4, cells 19 to 24. Figure 11 illustrates this scheme, where Fig. 11a shows Configuration 1, and Fig. 11b applies the same numbering to Configurations 2 and 3 to maintain consistency.

#### Single serpentine tube

**Fig. 12:**
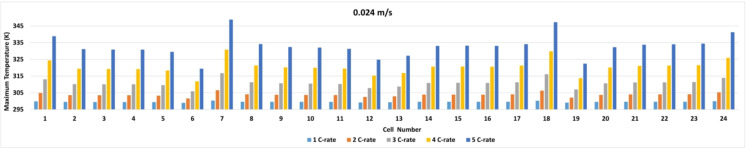
Battery maximum temperatures for Configuration 1 (serpentine flow) at 0.024 m/s under different C-rates.

The serpentine design shows pronounced temperature gradients along the flow direction. Cells near the inlet remain cooler, whereas outlet-side cells run hotter as heat accumulates downstream. Figure 12 reports the temperatures of 24 Li-ion cells at 0.024 $\:m/s$ for Configuration 1, which routes a single serpentine tube through the pack across 1 C to 5 C. It is clear from the figure that cell temperature rises with C-rate, and this is due to the increased internal heat generation. Above 3 C the temperature rise becomes steep. Cells later in the path reach higher maximum temperature because of the cumulative heating and declining local heat transfer. This highlights a core limitation of serpentine routing: upstream cells receive colder coolant and cool more effectively than downstream cells as the coolant warms. It is also worth noting that contact interfaces, especially the TIM and the battery-aluminum channel contact are vital for reducing thermal resistance. To study this effect, uneven contact was deliberately modeled at selected cells (e.g., cells 7 and 8). These findings show that both the cooling Configuration and battery contact quality strongly affect pack-level temperature.

The serpentine-cooled pack exhibited a strong dependence of cell temperature on discharge rate. At 1 C (3 $\:A$), the hottest cell (7) reached 27.2 $\:^\circ\:C$, while the coolest inlet-side cell (6) was at 25.8 $\:^\circ\:C$, giving a gradient of about 1.4 $\:^\circ\:C$. At 2 C (6 A), the maximum rose to 33.4 $\:^\circ\:C$, representing a 6.2 $\:^\circ\:C$ increase over 1 C, while the inlet-side cell (6) rose to 28.5 $\:^\circ\:C$, an increment of 2.7 $\:^\circ\:C$. At 3 C (9 $\:A$), the peak temperature jumped to 43.6 $\:^\circ\:C$, which is 10.2 $\:^\circ\:C$ higher than 2 C, while inlet cells increased to 32.7 $\:^\circ\:C$, a rise of 4.2 $\:^\circ\:C$. At 4 C (12 $\:A$), the maximum reached 57.7 $\:^\circ\:C$, showing a further 14.1$\:\:^\circ\:C$ rise compared to 3 C, while inlet cells reached 38.7 $\:^\circ\:C$, an additional 6.0 $\:^\circ\:C$ increase. At 5 C (15 $\:A$), the downstream cell temperature spiked to 75.7 $\:^\circ\:C$, marking a total rise of nearly 48.5 $\:^\circ\:C$ relative to 1 C, while the inlet-side cells increased more moderately to 46.3 $\:^\circ\:C$, or 20.5 $\:^\circ\:C$ above 1 C. These results indicate that temperature gradients across the pack widen sharply with increasing C-rate, from about 1.4 $\:^\circ\:C$ at 1 C to nearly 30 $\:^\circ\:C$ at 5 C, with downstream cells such as 7 and 18 consistently hotter due to accumulated heating, and local imperfections in thermal contact further confirming that both cooling Configuration and interface quality critically affect pack-level thermal performance.

The results validate Configuration 1 as a baseline but expose thermal limits under high loads and uneven contacts. Configuration 1 shows acceptable performance up to 3 C, but at higher C-rates it suffers from excessive temperatures. These findings motivate improved designs for EV applications where safety and uniform temperature are critical.

**Fig. 13:**
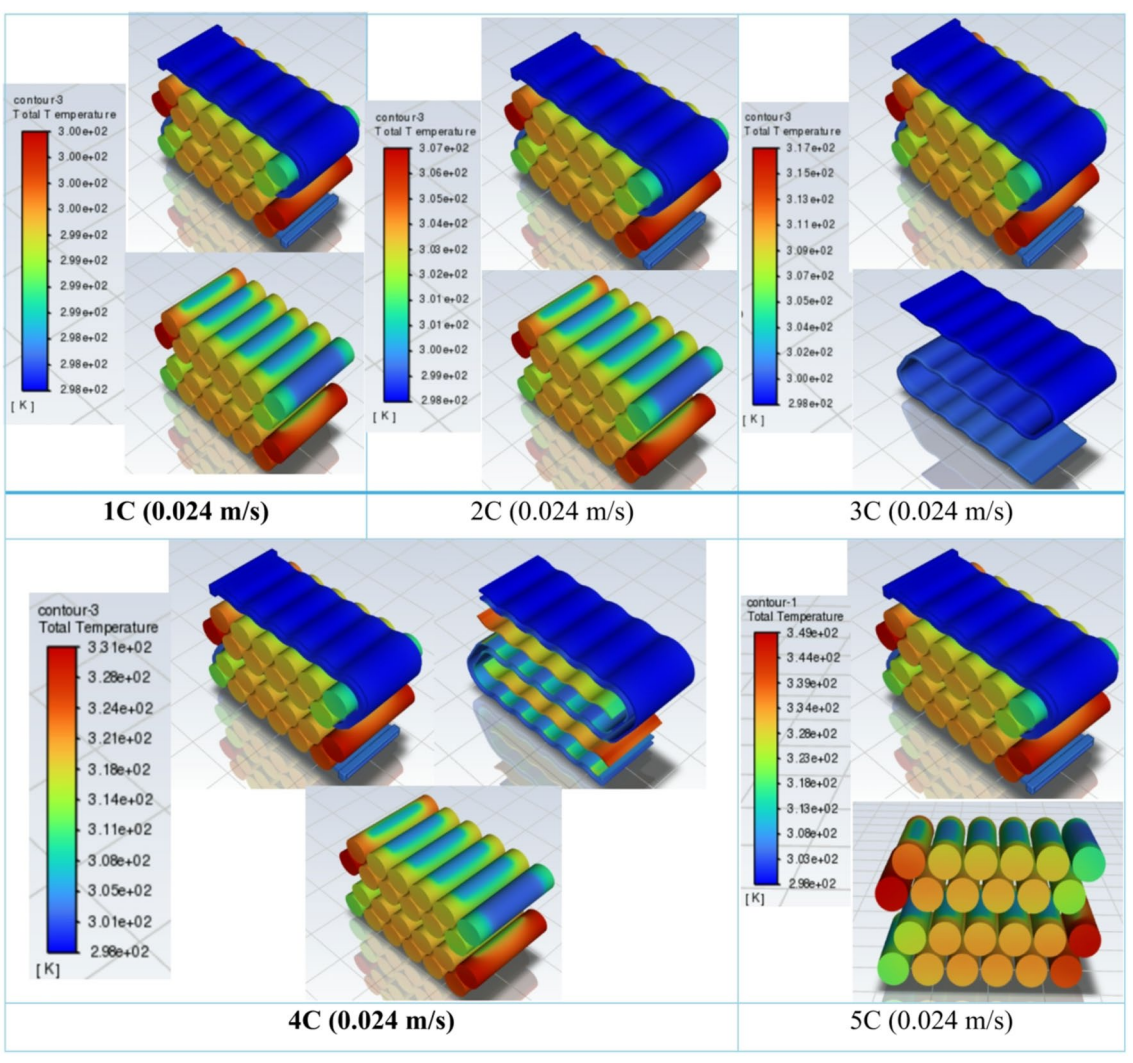
Temperature contour plots for Configuration 1 (serpentine channel) at a constant coolant velocity of 0.024 m/s under increasing discharge rates from 1 C to 5 C.

Figure 13 shows the temperature contours of the battery packs for Configuration 1 across the 24 cells at 0.024 m/s over discharge rates from 1 C to 5 C. Raising the C-rate increases internal heat generation, elevating T_max_ and broadening pack-wide thermal gradients. At 1 C, temperatures remain relatively uniform, with all cells below 27 $\:\circ ^C$. At 3 C, localized heating emerges, especially in mid and end path cells (7 and 18) attributed to insufficient contact area. At 5 C, a clear inlet to outlet gradient appears: T_max_ exceeds 75 $\:^\circ C$, driven by the warming unidirectional flow and the limited contact area of some cells. For instance, cell 7 reaches 75.7 $\:\circ ^C$ and cell 18 exceeds 74.1 $\:\circ ^C$, confirming a nonuniform profile. These results show that, despite adequate bulk velocity, serpentine routing imposes sequential cooling and thermal stacking, motivating parallel or counter-flow layouts in later sections.

#### Three parallel tubes

**Fig. 14:**
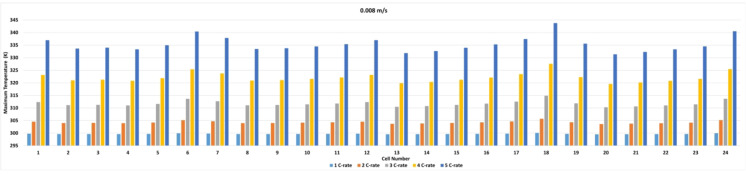
Temperature maximum distribution for Configuration 2 (parallel flow) at 0.008 m/s per channel.

The parallel design yields better temperature uniformity by cooling multiple cells simultaneously. In Configuration 2, illustrated in Fig. 2b and visualized in Fig. 14, three parallel cooling tubes are inserted between the battery rows, each receiving an inlet velocity of 0.008 $\:m/s$, giving the same total flow rate (0.024 $\:m/s$) as the single serpentine channel. Despite the reduced velocity per channel, the parallel distribution limits cumulative heat absorption along any one path and extracts heat simultaneously from each row, thereby reducing fluid-side resistance and preventing the inlet-to-outlet buildup observed in Configuration 1. This is evident in Fig. 14; the maximum cell temperature drops across all C-rates compared to Configuration 1 at the total flow rate. Quantitative results confirm this advantage: at 1 C, both systems show similar peak temperatures (~ 27 $\:^\circ\:C$), but the parallel layout narrows the gradient from 1.4 $\:^\circ\:C$ to 0.6 $\:^\circ\:C$. At 2 C, T_max_ falls from 33.4 $\:^\circ\:C$ (serpentine) to 32.6 $\:^\circ\:C$ (parallel) while ΔT drops from 4.9 $\:^\circ\:C$ to 2.1 $\:^\circ\:C$. At 3 C, parallel cooling reduces T_max_ by 1.9 $\:^\circ\:C$ and halves the gradient (10.9 $\:^\circ\:C$ → 4.5 $\:^\circ\:C$). At 4 C, the difference becomes more pronounced, with T_max_ reduced from 57.7 $\:^\circ\:C$ to 54.4 $\:^\circ\:C$ and ΔT cut from 19.0 $\:^\circ\:C$ to 8.0 $\:^\circ\:C$. Finally, at 5 C, the serpentine pack overheats to 75.7 $\:^\circ\:C$ with a 29.4 $\:^\circ\:C$ gradient, while the parallel pack remains at 70.6 $\:^\circ\:C$ with only a 12.4 $\:^\circ\:C$ gradient. Former hotspots (cells 7, 12, 18, and 24) now fall within a much narrower range, confirming that parallel cooling not only lowers absolute temperatures but also significantly enhances thermal uniformity across the pack. Expressed as a reduction, the parallel layout narrows the inter-cell gradient by 57%, 57%, 59%, and 58% at 2 C, 3 C, 4 C, and 5 C respectively relative to the serpentine design, while lowering peak temperature by up to 5.1 $\:^\circ\:C$ at 5 C.

Surface contact between the cells and cooling plates remains critical. Cells with optimized contact and uniform TIM application, such as in cells 2–5, 8–11,13–17 and 20–23, show better thermal performance. In contrast, degraded contacts, for example, cells 6,7, 21, and 24 produce localized temperature spikes, even in the improved Configuration. Thus, increasing the contact area is as important as channel-level design for effective BTMS.

**Fig. 15:**
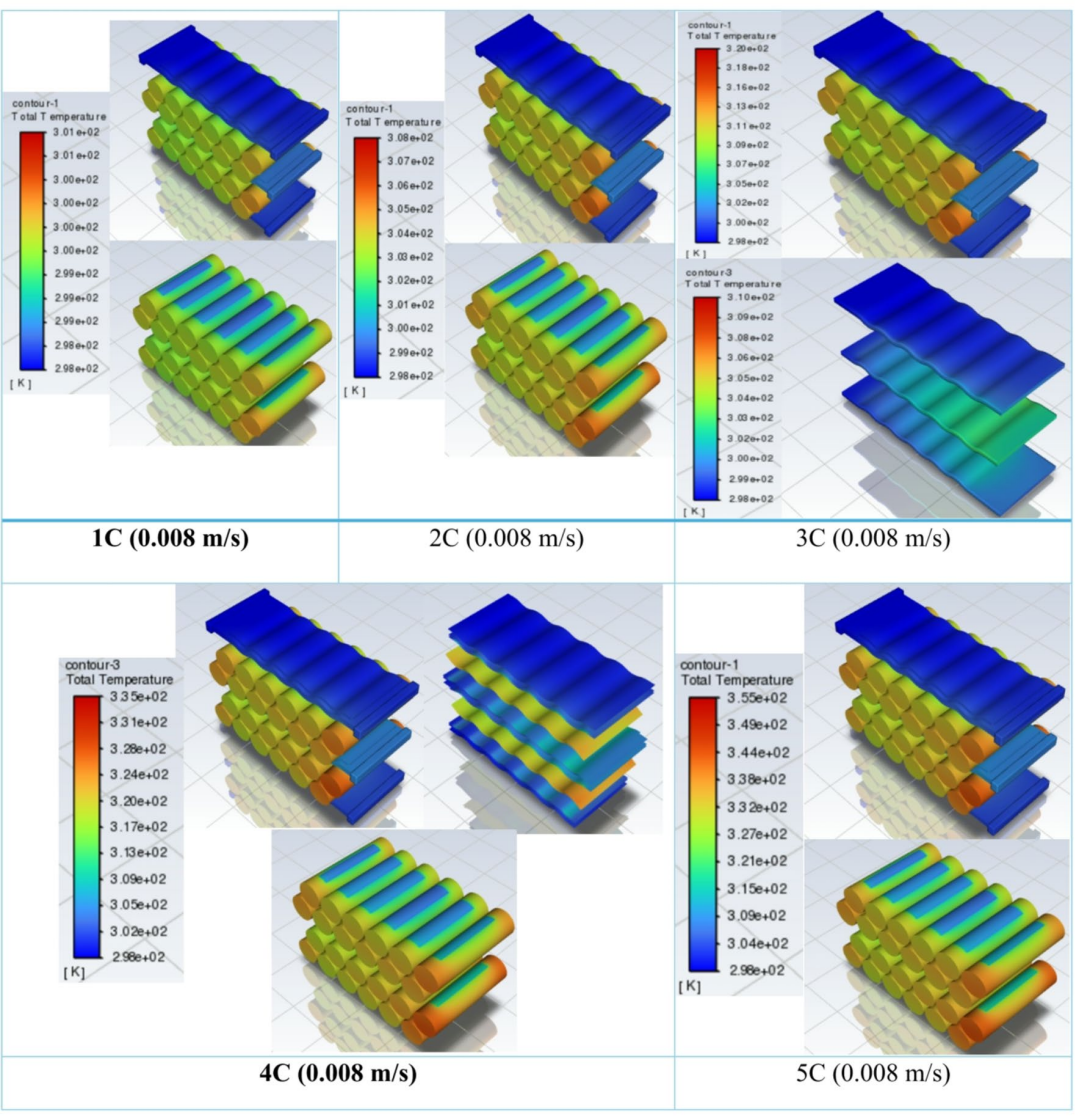
Temperature contour plots of Configuration 2 (three parallel cooling channels) under 1 C to 5 C discharge rates at a constant coolant velocity of 0.008 m/s per channel.

Figure 15 illustrates the temperature contours for Configuration 2 under discharge rates ranging from 1 C to 5 C at a coolant velocity of 0.008 m/s per channel. In this setup, the coolant enters three separate inlets, each serving a row of six cells, which shortens the flow path and enables simultaneous heat extraction. As a result, the overall cell temperatures are markedly reduced compared to Configuration 1. For example, at 1 C the pack operated between 26.4 $\:^\circ\:C$ (cell 22) and 27.0 $\:^\circ\:C$ (cell 18), giving a narrow spread of only 0.6 $\:^\circ\:C$. At 2 C, temperatures rose to 30.5–32.6 $\:^\circ\:C$, with a spread of 2.1 $\:^\circ\:C$, nearly half the 4.9 $\:^\circ\:C$ variation observed in Configuration 1. At 3 C, the hottest cell reached 41.7 $\:^\circ\:C$ (cell 18) while the coolest remained at 37.2 $\:^\circ\:C$ (cell 22), giving a ΔT of 4.5 $\:^\circ\:C$—less than half the 10.9 $\:^\circ\:C$ gradient in serpentine cooling. At higher loads, the benefit becomes even more apparent: at 4 C the parallel design limited T_max_ to 54.4 $\:^\circ\:C$, compared to 57.7 $\:^\circ\:C$. Finally, at 5 C the pack peaked at 70.6 $\:^\circ\:C$ (cell 18) while the lowest was 58.2 $\:^\circ\:C$ (cell 22). Despite these improvements, local variations remain evident: corner or edge cells such as 6, 7, and 24 recorded 3–6 $\:^\circ\:C$ higher than the coolest regions, underscoring the influence of thermal interface quality and channel proximity. Overall, Configuration 2 delivers superior heat mitigation, with temperature spreads consistently narrowed by 40–60% across all C-rates relative to Configuration 1, confirming the effectiveness of parallel flow distribution in BTMS.

#### Counter-flow tube

**Fig. 16:**
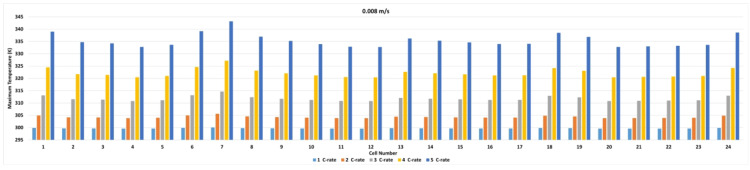
Temperature maximum distribution for Configuration 3 (parallel + counter-flow) at 0.008 m/s.

The counter-flow design achieved the best thermal performance, minimizing temperature rise and improving uniformity. In Configuration 3, depicted in Fig. 2c and quantitatively represented in Fig. 16, two parallel channels and one counter-flow tube operate at a flow velocity of 0.008 m/s, as in Configuration 2. This maintains the same total inlet flow rate as Configuration 1, enabling direct comparison at matched flowrate.

The counter-flow feature reverses coolant direction over part of the pack, balancing inlet-outlet gradients and mitigating downstream heat accumulation typical of unidirectional layouts. Compared to Configuration 1, Configuration 3 shows lower T_max_ and tighter pack-level uniformity through divided and redirected flow. Compared to Configuration 2, the difference in peak temperature and thermal uniformity over each cell is relatively minor, primarily because the number of cells per row is limited, reducing the total heat accumulation in each stream. However, Configuration 3 offers marginally better pack-level thermal uniformity which will be analyzed in more detail in Sect. 5.3.

At 1 C (3 A), Configuration 3 maintained a maximum temperature of 27.0 $\:^\circ\:C$. At 3 C (9 $\:A$), the maximum temperature rose to 41.5 $\:^\circ\:C$; this corresponds to about 4.8% lower T_max_ than Configuration 1 (≈ 43.6 $\:^\circ\:C$). Under the most severe condition of 5 C (15 $\:A$), the pack reached a maximum of 70.0 $\:^\circ\:C$, which is 5.7 $\:^\circ\:C$ cooler than Configuration 1 (≈ 75.7 $\:^\circ\:C$) and 0.6 $\:^\circ\:C$ cooler than Configuration 2 (≈ 70.6 $\:^\circ\:C$). These results confirm that the counter-flow arrangement distributes heat extraction more evenly across the pack, limiting hotspot formation in downstream cells. The improvements over Configuration 2 are modest, primarily because the limited cells per row reduce heat accumulation per stream; nevertheless, Configuration 3 consistently provides the lowest T_max_ and ΔT across all C-rates and offers marginally better pack-level thermal uniformity which will be analyzed in more detail in Sect. 5.3.

Figure 16 shows a narrower spread of maximum temperature and more even gradients than other two Configurations. Critical cells such as 6, 12, 18, and 24 now lie closer to the pack mean, indicating effective temperature smoothing. Surface contact and TIM still govern local behavior; where contact is suboptimal, small anomalies persist, confirming the influence of interface resistance.

**Fig. 17:**
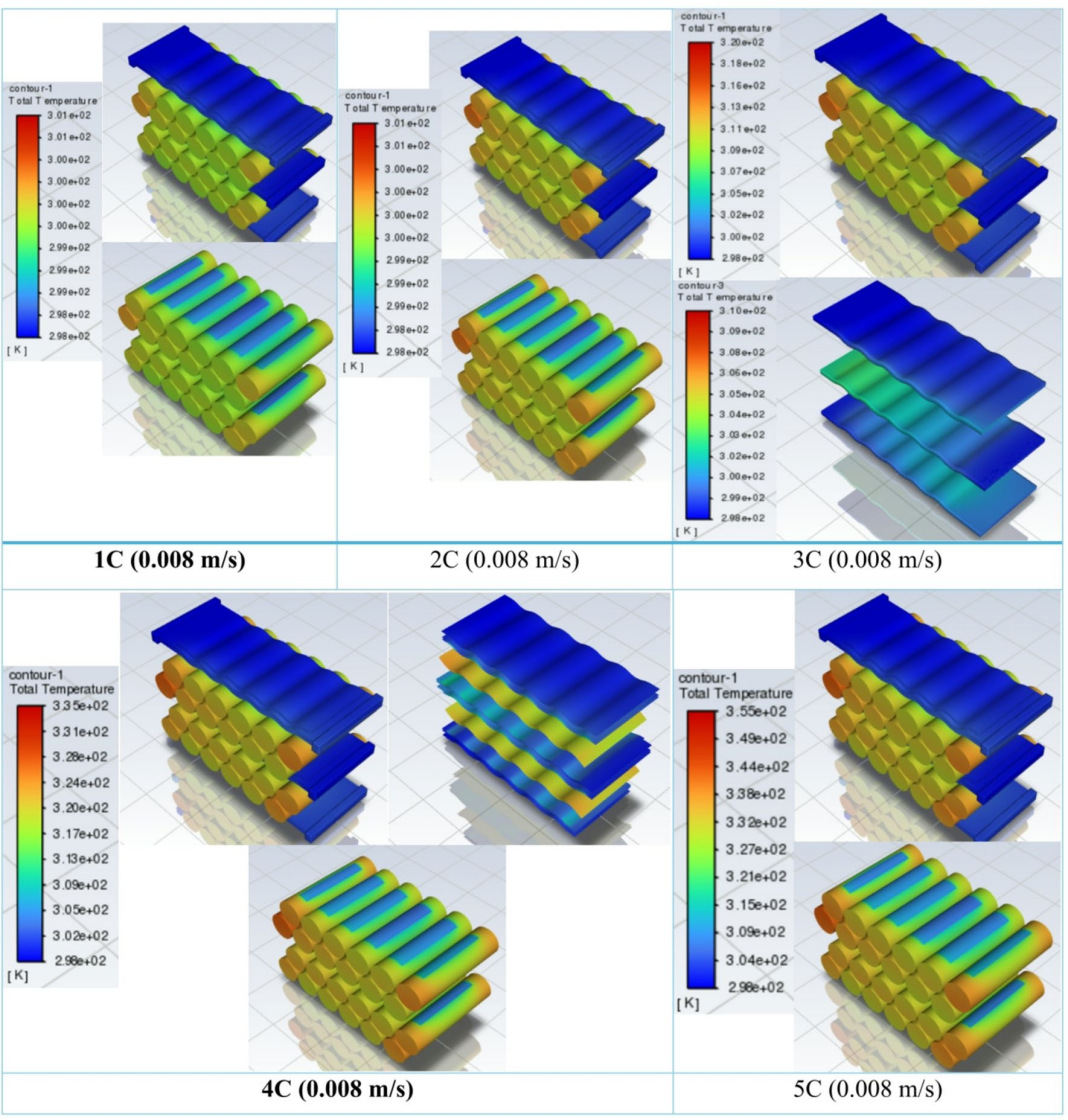
Temperature contour distribution of Configuration 3 (two parallel + one counter-flow channel) at 0.008 m/s coolant velocity per channel under discharge rates from 1 C to 5 C.

Figure 17 shows the temperature contours for Configuration 3, which employs two parallel channels and one counter-flow channel, under discharge rates ranging from 1 C to 5 C at a fixed coolant velocity of 0.008 $\:m/s$ per channel. As expected, higher C-rates increase the thermal load, raising cell temperatures across the pack. At 1 C, the pack temperature remained tightly clustered between 25.5 $\:^\circ\:C$ and 27.0 $\:^\circ\:C$ (ΔT = 1.5 $\:^\circ\:C$). At 3 C, Configuration 3 limited the maximum temperature to 41.5 $\:^\circ\:C$, around 2.1 $\:^\circ\:C$ cooler than Configuration 1 (≈ 43.6 $\:^\circ\:C$) and 0.3 $\:^\circ\:C$ cooler than Configuration 2 (≈ 41.7 $\:^\circ\:C$). At severe conditions of 5 C, Configuration 3 peaked at 70.0 $\:^\circ\:C$, which is 5.7 $\:^\circ\:C$ lower than Configuration 1 (≈ 75.7 $\:^\circ\:C$) and 0.6 $\:^\circ\:C$ lower than Configuration 2 (≈ 70.6 $\:^\circ\:C$). These results confirm that the counter-flow design effectively redistributes coolant, reducing hotspot formation and balancing inlet-outlet gradients. While the differences from Configuration 2 are modest in uniformity, Configuration 3 consistently achieves the lowest maximum temperatures and most symmetric distribution, particularly in mid-path and outlet-side cells, making it the most effective layout for pack-level thermal control.

Maximum cell temperature differs across Configurations, especially between Configuration 1 and the parallel/counter-flow layouts, highlighting distinct thermal behaviors and cooling effectiveness. In Configuration 1, the maximum temperature at 5 C occurred at cell 7 (75.7 $\:^\circ\:C$), with cell 18 at 74.1 $\:^\circ\:C$, illustrating sequential flow and downstream heat buildup. In Configuration 2, the pack maximum dropped to 70.6 $\:^\circ\:C$ at cell 18, a 6.7% reduction compared to Configuration 1. Notably, cell 7 cooled more strongly to 64.7 °C, corresponding to a 14.8% reduction, while cell 6 stabilized at 67.3 $\:^\circ\:C$. In Configuration 3, under the most severe condition of 5 C (15 $\:A$), the pack reached a maximum of 70.0 $\:^\circ\:C$ which is 5.7 $\:^\circ\:C$ cooler than Configuration 1 (≈ 75.7 $\:^\circ\:C$) and 0.6 $\:^\circ\:C$ cooler than Configuration 2 (≈ 70.6 $\:^\circ\:C$). Overall, both Configurations 2 and 3 significantly reduce peak temperatures compared to Configuration 1, with Configuration 2 yielding the coolest local hotspot at cell 7, but Configuration 3 achieving the lowest overall pack maximum and the most balanced temperature distribution. The persistent elevation at cell 6, attributed to degraded thermal contact, underscores the importance of applying TIM across the pack to ensure uniform heat dissipation.

**Fig. 18:**
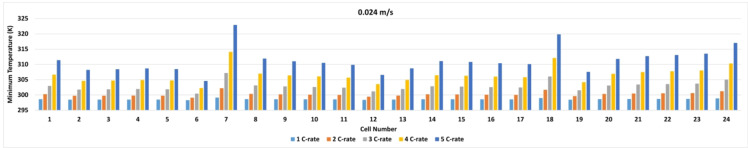
Battery minimum temperatures for Configuration 1 (serpentine flow) at 0.024 m/s under different C-rates.

**Fig. 19:**
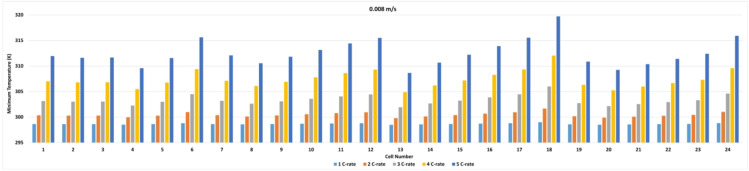
Temperature minimum distribution for Configuration 2 (parallel) at 0.008 m/s.

**Fig. 20:**
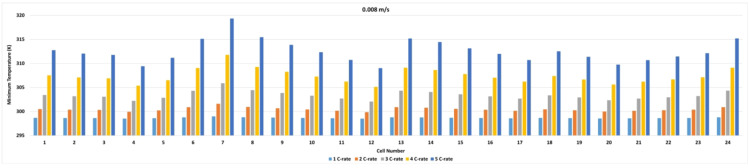
Temperature minimum distribution for Configuration 3 (parallel + counter-flow) at 0.008 m/s.

Figures 18, 19 and 20 present the minimum cell temperatures for Configurations 1–3 under discharge rates of 1 C to 5 C at a coolant velocity of 0.008 $\:m/s$ per channel. In Configuration 1, the minimum temperature increases from 25.1 $\:^\circ\:C$ at 1 C to 38.9 $\:^\circ\:C$ at 5 C, with cell 6 recording 31.3 $\:^\circ\:C$ as the lowest value at 5 C. In Configuration 2, the minimum rises from 25.5 $\:^\circ\:C$ at 1 C to 38.8 $\:^\circ\:C$ at 5 C, with cell 4 registering 36.4 $\:^\circ\:C$ at the highest load, representing a 5.1 $\:^\circ\:C$ increase compared to Configuration 1. In Configuration 3, T_min_ starts at 25.5 $\:^\circ\:C$ at 1 C and reaches 39.6 $\:^\circ\:C$ at 5 C, with cell 4 showing 36.3 $\:^\circ\:C$ at 5 C, very close to Configuration 2 but still slightly higher than Configuration 1. These results show that while all Configurations follow the expected rising trend of T_min_ with increasing C-rate, Configurations 2 and 3 maintain higher baseline minimum values compared to Configuration 1.

Degraded contacts cause deviations especially at boundary cells such as 6 and 18 highlighting the need for effective TIM layer. Configuration 3, which is presented in Fig. 20, shows the most balanced minimum temperature profile across the 24 cells. The reverse path dissipates heat and equalizes thermal exposure.

### Effect of coolant flow rate

**Fig. 21:**
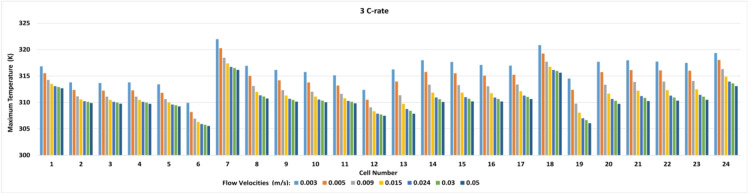
Maximum temperature distribution across all 24 cells at various coolant flow velocities (0.003–0.05 m/s) for Configuration 1 under 3 C-rate discharge.

**Fig. 22:**
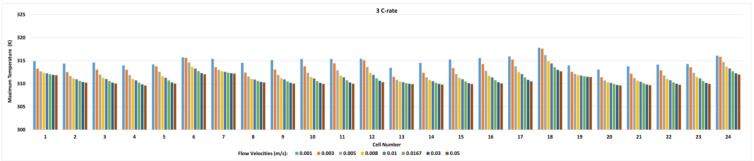
Maximum temperature distribution at different coolant flow velocities (0.001–0.05 m/s) for Configuration 2 under 3 C-rate discharge.

**Fig. 23:**
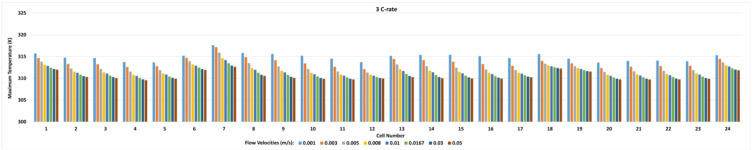
Maximum temperature variation with increasing coolant velocity (0.001–0.05 m/s) for Configuration 3 at 3 C-rate discharge.

Across all Configurations, maximum cell temperature decreases as coolant velocity increases, reflecting stronger convective cooling. However, this cooling enhancement diminishes beyond 0.008 $\:m/s$ most evident in Configuration 2 and Configuration 3. In Configuration 1, cell 7 temperature drops by 1.8% (5.81 $\:^\circ\:C$) and cell 18 temperature drops by 1.63% (5.22 $\:^\circ C)\:$when velocity rises from 0.003 to 0.05 $\:m/s$. In Configuration 2, cell 7 temperature drops by 1% (3.18 $\:^\circ C)$ and cell 18 temperature drops by 1.61% (5.11 $\:^\circ\:C)$ when velocity increases from 0.008 to 0.05 m/s. In Configuration 3, the temperatures of cells 7 and 18 decrease by 1.57% (4.97 $\:^\circ\:C$) and by 3.32 $\:^\circ\:C$, respectively when coolant velocity increases from 0.008 to 0.05 $\:m/s$, respectively. While all designs benefit from higher velocity, improvement slows markedly after 0.008 $\:m/s$ in Configuration 2 and 3, indicating a cooling saturation region where further velocity increases yield only marginal reductions in T_max_. Figures 21, 22 and 23 compare the maximum temperature distribution of the 24-cell pack under 3 C discharge for Configurations 1–3, showing the effect of varying coolant velocity from 0.001 to 0.05 $\:m/s$; as the flow rate increases, all Configurations exhibit a reduction in T_max_, with Configuration 3 maintaining the lowest values, followed by Configuration 2, while Configuration 1 consistently records the highest peak temperatures.

### Cell uniformity

In battery thermal analysis, it is essential to distinguish between inter-cell thermal uniformity (often referred to as Thermal Uniformity over the Entire Pack) and intra-cell thermal uniformity (also called Thermal Uniformity over Each Cell), as they address different thermal challenges and serve distinct engineering purposes.

#### Inter-cell thermal uniformity (thermal uniformity over the entire pack)

Inter-cell thermal uniformity evaluates the temperature consistency among all cells in the pack. Minimizing deviations is crucial for safe, efficient operation because large temperature differences accelerate aging, cause uneven capacity fade, and degrade performance. The average temperature of each of the 24 cells was analyzed at 3 C for three cooling Configurations. The metrics used to assess uniformity included the mean temperature, standard deviation, CV, and maximum temperature difference (ΔT_inter_).

Table 7 reports the calculated values for these parameters. Configuration 1, characterized by a high coolant velocity (0.024 $\:m/s$), exhibited the poorest inter-cell uniformity with a standard deviation of 2.406 $\:K$ and ΔT_inter_ of 11.04 $\:K$. This large spread is attributed to the sharp temperature rise in certain cells (e.g., Cell 7) despite the moderate average pack temperature. In contrast, Configurations 2 and 3, which utilized parallel and counter cooling, showed improved uniformity. Configuration 3 achieved the best performance with a coefficient of variation of 0.32% and a ΔT_inter_ of only 4.14 $\:K$. Relative to Configuration 1 (CV = 0.78%, σ = 2.41 $\:K$), Configuration 3 reduced the coefficient of variation by 59% (to 0.32%) and the standard deviation by 58% (to 1.0 $\:K$) and cut ΔT_inter_ by 62.5% (11.04 $\:K$ to 4.14 $\:K$); Configuration 2 achieved comparable gains (CV 0.36%, σ = 1.11 $\:K$). This suggests that parallel flow designs, even at lower flow rates, can more effectively equalize cell temperatures than single-loop serpentine designs at higher velocities. These findings highlight the importance of not only coolant speed but also flow distribution and Configuration geometry in maintaining thermal balance across the pack.

**Table 7:** Inter-cell thermal uniformity metrics for three cooling Configurations at 3 C discharge rate.

Metric	Configuration 1	Configuration 2	Configuration 3
Mean Cell Temperature ($\:\boldsymbol{K}$)	308.51	309.35	309.36
Standard Deviation ($\:\boldsymbol{K}$)	2.41	1.11	1.00
Coefficient of Variation (%)	0.78	0.36	**0.32**
Max Cell Temp Difference ΔT_inter_ ($\:\boldsymbol{K}$)	11.04	4.65	**4.14**

#### Intra-cell thermal uniformity (thermal uniformity over each cell)

Intra-cell thermal uniformity refers to the temperature difference within each individual cell, typically represented by the difference between the maximum and minimum temperatures across the cell’s surface. This metric is critical because internal temperature gradients can induce mechanical stress, localized aging, electrode degradation, and thermal runaway risk, especially under high C-rate operations. The intra-cell thermal non-uniformity for each of the 24 cylindrical cells was computed under three different cooling Configurations at a 3 C discharge rate. The temperature difference (ΔTₖ) for each cell was derived from simulation outputs. Table 8 compares the intra-cell thermal uniformity across the three cooling Configurations. Configuration 1 contains the single cell with the smallest internal gradient (ΔTₖ = 5.48 K), but simultaneously the widest range (4.66 K) and the highest standard deviation (1.09 K), indicating that this low minimum is an outlier rather than evidence of uniform heat dissipation. Configuration 2 delivered the best uniformity, with the lowest standard deviation, 0.47 $\:K$, and coefficient of variation 5.6%, this represents a 57% reduction in intra-cell standard deviation (1.09 K to 0.47 K) and a 60% reduction in coefficient of variation (13.9% → 5.6%) relative to Configuration 1, showing that distributing flow across separate paths enhances cell heat extraction. Configuration 3 showed slightly higher variability, likely from flow alignment differences. These results confirm that the flow distribution and channel Configuration, not flow rate alone, govern intra-cell uniformity.

**Fig. 24:**
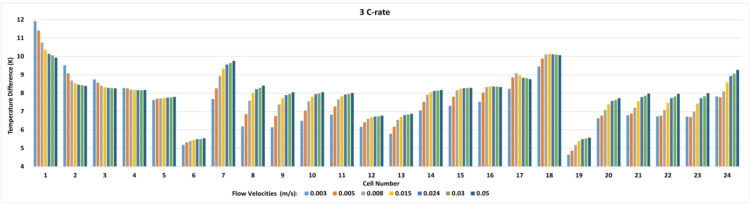
Temperature difference (ΔT) distribution over each cell in Configuration 1 (serpentine cooling) under a 3 C discharge.

**Fig. 25:**
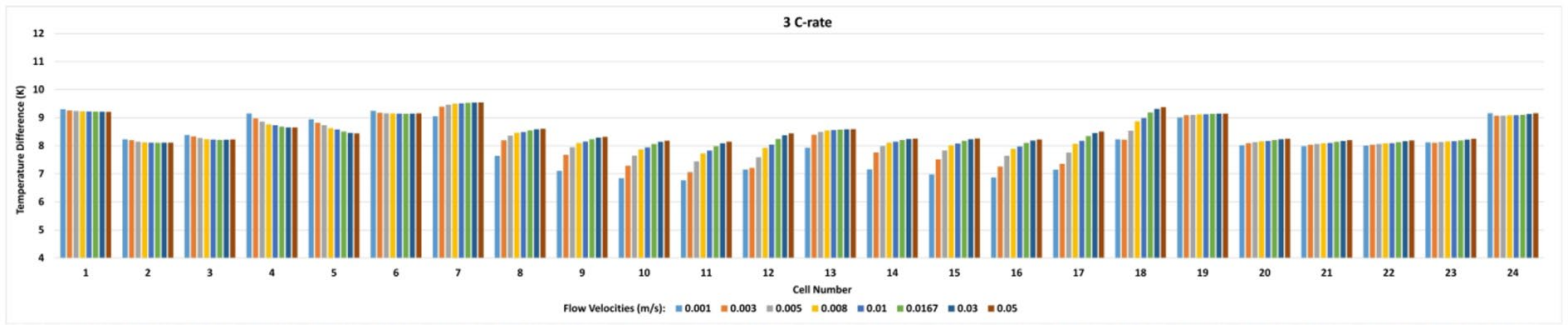
Thermal uniformity over each cell in Configuration 2 (Parallel cooling) under a 3 C discharge.

**Fig. 26:**
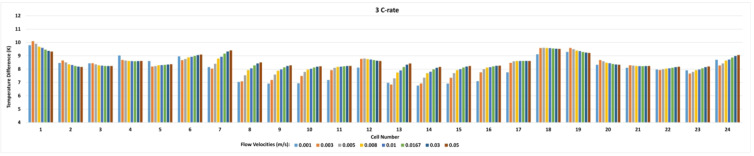
Thermal uniformity over each cell in Configuration 3 (Counter cooling) under a 3 C discharge.

**Fig. 27:**
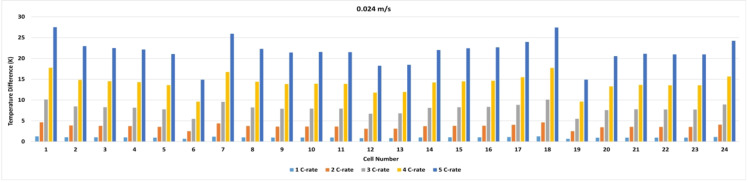
Temperature difference (ΔT) distribution over each cell in Configuration 1 (serpentine cooling at 0.024 m/s) under different discharge rates.

**Fig. 28:**
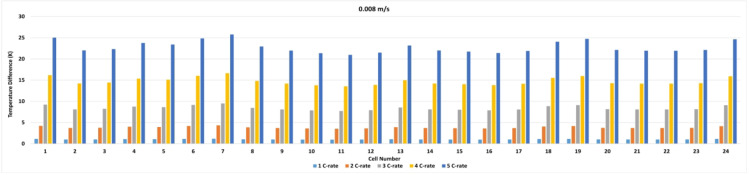
Temperature difference (ΔT) distribution over each cell in Configuration 1 (serpentine cooling at 0.024 m/s) under different discharge rates.

**Fig. 29:**
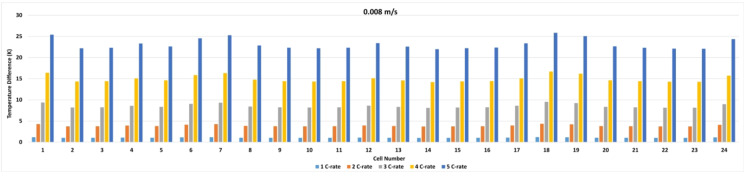
Thermal uniformity over each cell in Configuration 3 (Counter cooling at 0.008 m/s) under different discharge rates.

Figures 24, 25, 26, 27, 28, 29 visualize intra-cell thermal uniformity, expressed as the temperature difference (ΔT) within each of the 24 cells across the three studied Configurations under fixed and varying discharge conditions. In Fig. 24 (Configuration 1), serpentine cooling led to significant variation in ΔT among the cells. Some cells, cells 1 and 18, exhibited ΔT values exceeding 10 K, while others, cells 6 and 19, showed much lower values (~ 5.5 K). This reflects serial flow in which upstream cells are colder and downstream cells are warmer, creating uneven heat removal and a high standard deviation. Configuration 2 (Fig. 25) shows tighter clustering around the mean. Parallel distribution supplies each row simultaneously, reducing the coefficient of variation to 5.6%. Configuration 3 (Fig. 26) performs slightly worse compared to Configuration 2 but better than Configuration 1. Counter-flow reduces through-pack gradients but lacks the full symmetry and pressure balance of parallel channels. Cells near the flow reversal region remain mildly higher ΔT.

**Table 8:** Intra-cell thermal uniformity comparison across three cooling Configurations (ΔT = T_max_ – T_min_ per cell).

Metric	Config 1 (0.024 m/s)	Config 2 (0.008 m/s)	Config 3 (0.008 m/s)
Mean Temp Difference (K)	7.85	8.37	8.34
Max Temp Diff (K)	10.14	9.50	9.68
Min Temp Diff (K)	5.48	7.73	7.69
Range ΔT (K)	4.66	1.77	1.99
Standard Deviation (K)	1.09	**0.47**	0.54
Coefficient of Variation %	13.9%	**5.6%**	6.5%

### Pressure drop analysis

To evaluate the hydrodynamic performance of the three cooling Configurations, pressure drop (∆P) was evaluated across a range of coolant flow velocities (0.001 to 0.05 $\:m/s$) under a fixed 3 C discharge condition. The total pressure loss in each Configuration reflects the pumping power demand, which directly impacts the energy efficiency and operational cost of the BTMS.

**Fig. 30:**
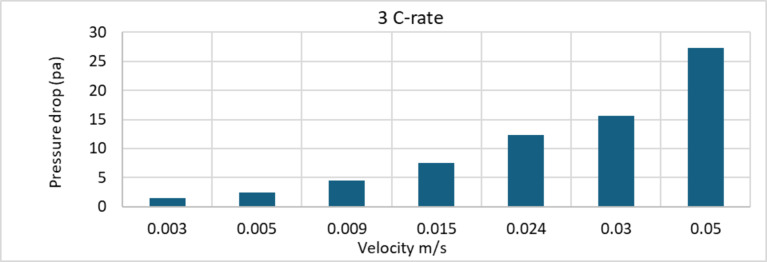
Pressure drop across the single serpentine cooling channel (Configuration 1) under a 3 C discharge rate.

In Configuration 1, the coolant travels sequentially through all 24 cells, resulting in a longer flow path and higher hydraulic resistance. As shown in Fig. 30, the pressure drop increased nonlinearly with velocity, reaching a maximum of 27.34 $\:Pa$ at 0.05 $\:m/s$. Even at moderate velocities, such as 0.024 $\:m/s$, the pressure drop was 12.34 $\:Pa$, indicating significant resistance due to the serpentine geometry. This Configuration requires higher pumping power, especially at elevated flow rates, making it less energy-efficient, despite its simplicity.

**Fig. 31:**
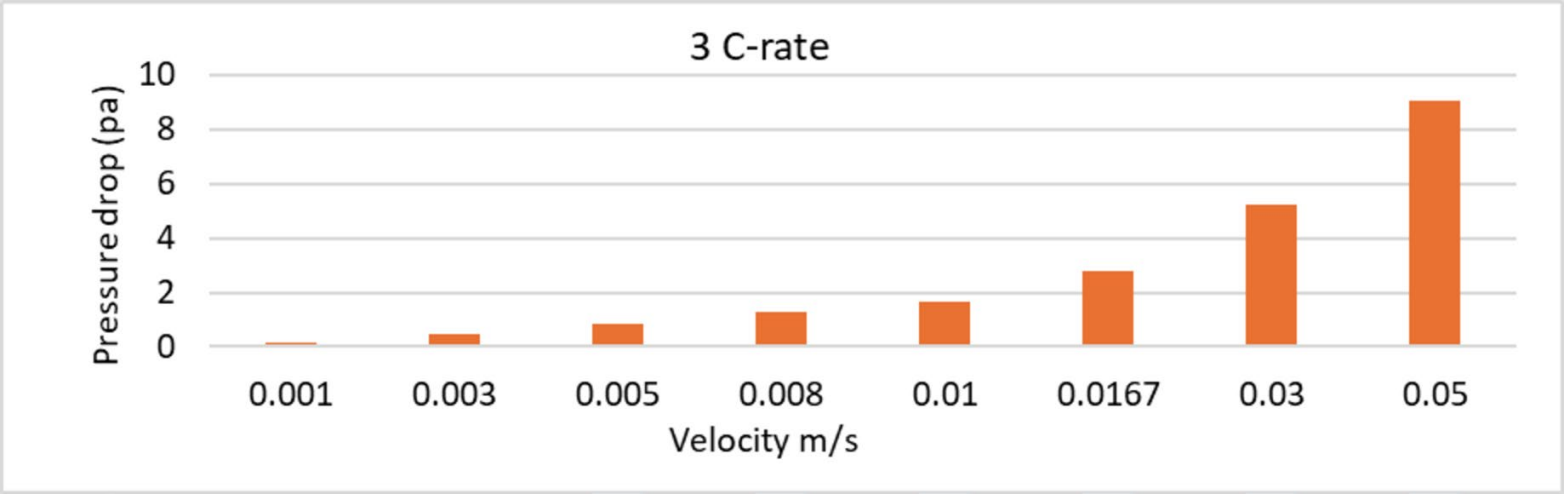
Pressure drop comparison for Configuration 2 (three parallel channels) and Configuration 3 (two parallel + one counter-flow channel) under a 3 C discharge rate.

In Configuration 2 (three parallel channels), the coolant divides among three shorter parallel paths, significantly reducing the flow length and frictional losses per channel. At 0.05 $\:m/s$, the average pressure drop across the three channels was approximately 9.07 $\:Pa$, which is 33% lower than Configuration 1 at the same normalized flow rate. At 0.01 $\:m/s$, pressure drop remained below 1.65 $\:Pa$, offering energy-efficient performance at typical EV cooling conditions. This demonstrates that parallelization helps achieve better cooling with less hydraulic penalty. Configuration 3 behaves similarly to Configuration 2, as all flow paths are nearly identical in geometry. The maximum pressure drop at 0.05 $\:m/s$ was around 9.07 $\:Pa$ (Fig. 31).

### Pumping power and thermal-hydraulic efficiency

Pressure drop alone does not capture the energy penalty of a cooling layout; the relevant quantity is the pumping power, $\:{P}_{\mathrm{pump\:}}={\Delta\:}P\cdot\:Q$. Because the parallel and serpentine layouts distribute flow differently, pumping power was evaluated at a matched total volumetric flow rate, so that differences reflect channel topology rather than the supplied flow. Table 9 reports $\:{\Delta\:}P,{Q}_{\mathrm{total\:}}$, and $\:{P}_{\mathrm{pump\:}}$ for the three Configurations across matched operating points.

**Table 9:** Pumping power and thermal-hydraulic performance of the three Configurations at matched total volumetric flow rates.

$\:\mathrm{M}\mathrm{a}\mathrm{t}\mathrm{c}\mathrm{h}\mathrm{e}\mathrm{d}\:{\boldsymbol{Q}}_{\mathrm{total\:}}\:(\:{\mathbf{m}}^{3}/\mathbf{s}$)	Config. 1			Config. 2 & 3			Pumping Power Reduction
	Velocity, $\:v$ (m/s)	Pressure Drop, $\:{\Delta\:}P\:\left(\mathrm{P}\mathrm{a}\right)$	Pumping Power, $\:{P}_{\mathrm{pump\:}}\left(\mu\:\mathrm{W}\right)$	Velocity, $\:{v}_{\mathrm{br\:}}$ (m/s)	Pressure Drop, $\:{\Delta\:}P\:\left(\mathrm{P}\mathrm{a}\right)$	Pumping Power, $\:{P}_{\mathrm{pump\:}}\left(\mu\:\mathrm{W}\right)$	
$\:6.0\times\:{10}^{-7}$	0.003	1.3	0.78	0.001	0.2	0.12	$\:85\mathrm{\%}$
$\:1.8\times\:{10}^{-6}$	0.009	4.3	7.74	0.003	0.5	0.90	$\:88\mathrm{\%}$
$\:3.0\times\:{10}^{-6}$	0.015	7.4	22.2	0.005	0.8	2.40	$\:89\mathrm{\%}$
$\:4.8\times\:{10}^{-6}$ (design point)	0.024	12.34	59.2	0.008	1.3	6.24	$\:89\mathrm{\%}$
$\:6.0\times\:{10}^{-6}$	0.030	15.6	93.6	0.010	1.65	9.9	$\:89\mathrm{\%}$

At the $\:0.024\text{}\mathrm{m}/\mathrm{s}$ reference flow, Configuration 1 demands $\:59.2\:\mu\:W$ of pumping power, whereas Configurations 2 and 3 require only $\:6.24\:\mu\:W$ - an $\:89\mathrm{\%}$ reduction because splitting the flow into three short parallel branches lowers per-channel frictional loss. Crucially, this reduction is achieved while Configurations 2 and 3 simultaneously lower the peak pack temperature, so the parallel layouts outperform the serpentine layout on both cooling performance and energy cost. Defining a thermal-hydraulic efficiency $\:{\eta\:}_{\mathrm{th-h\:}}={\stackrel{\prime }{Q}}_{\mathrm{removed\:}}/{P}_{\mathrm{pump\:}}$ (heat removed per unit pumping power), Configurations 2 and 3 are therefore nearly an order of magnitude more efficient at equal total flow. The absolute pumping powers are small owing to the low velocities and compact cross-section, but the $\:\sim\:9\times\:$ relative difference scales directly with pack count and fleet size, where parasitic pumping load erodes vehicle range.

Table 10 consolidates the key performance indices for the three Configurations at 3 C. Relative to the serpentine baseline (Configuration 1), both parallel-based layouts lower peak temperature by ~ 2 $\:^\circ\:C$, more than halve the inter-cell gradient (11.04 $\:K$ → 4.65 and 4.14 $\:K$), and cut the coefficient of variation from 0.78% to 0.36% and 0.32%. Configuration 3 attains the lowest Tmax (41.5$\:\:^\circ\:C$), narrowest inter-cell ΔT (4.14 $\:K$, a 62.5% reduction), and best inter-cell uniformity (CV = 0.32%), while Configuration 2 shows marginally better intra-cell uniformity (σ = 0.47 vs. 0.54 $\:K$). Both achieved these gains at 6.24 $\:\mu\:W$—an 89% pumping-power reduction versus Configuration 1 (59.2 $\:\mu\:W$), giving a nearly order-of-magnitude higher thermal-hydraulic efficiency (~ 9×). Overall, flow distribution and channel topology, not coolant velocity alone, govern both thermal and energy performance: Configuration 3 offers the most balanced result, while Configuration 2 captures most of the benefit at lower complexity.

**Table 10:** The key performance indices for the three Configurations at 3 C discharge.

Index (at 3 C)	Config 1	Config 2	Config 3
Tmax (°C)	43.6	41.7	41.5
Inter-cell ΔT (K)	11.04	4.65	4.14
Inter-cell CV (%)	0.78	0.36	0.32
Intra-cell σ (K)	1.09	0.47	0.54
Pumping power (µW)	59.2	6.24	6.24
η_th-h (relative)	1.0×	~ 9×	~ 9×

## Conclusion

A combined numerical and experimental study compared three liquid-cooling layouts for an NMC Li-ion module: serpentine (Configuration 1), parallel (Configuration 2), and hybrid parallel/counter-flow (Configuration 3). A four-cell test rig validated the CFD model to within ± 1.6 $\:K$ (relative deviation below 0.6%). Key findings are summarized as follows:

- Peak temperature: Configuration 1 was the hottest (75.7 $\:^\circ\:C$ at cell 7 and 74.1 $\:^\circ\:C$ at cell 18). Configuration 2 lowered the peak to 70.6 $\:^\circ\:C$ (− 6.7% versus Configuration 1), and Configuration 3 gave the lowest peak (− 7.6% versus Configuration 1), reducing cell 18 by 11.8%.
- Inter-cell uniformity: Configuration 3 was best, with σ = 1.00 $\:K$, CV = 0.32% and the narrowest spread (ΔT = 4.14 $\:K$) – a 62.5% reduction versus serpentine (11.04 $\:K$) and 11% versus parallel (4.65 $\:K$).
- Intra-cell uniformity: Configuration 2 was best (σ = 0.47 $\:K$, CV = 5.6%).
- Hydraulic cost: Configuration 1 incurred the highest pressure drop (27.34 $\:Pa$) and pumping power (59.2 $\:\mu\:W$); Configurations 2 and 3 cut pumping power to ≈ 6.24 $\:\mu\:W$ (− 89% at matched flow), giving a nearly ten-fold higher thermal–hydraulic efficiency.
- Beyond cooling layout, the study demonstrated that surface contact quality and the use of TIMs are equally decisive in preventing localized hotspots, particularly in boundary and mid-path cells. Flow rate analysis further revealed diminishing returns beyond moderate velocities (≈ 0.008–0.024 $\:m/s$), identifying a saturation region where higher pumping effort yields marginal improvements.

Overall, while Configuration 1, although simple, suffered from excessive thermal gradients and pressure loss. Configuration 2 excelled in intra-cell uniformity and hydrodynamic efficiency as it provides a balanced solution for efficient heat transfer and minimal pumping losses. Configuration 3 demonstrated the best thermal balance as slightly improves thermal uniformity but at the cost of added complexity, Future designs should incorporate advanced flow layouts, TIM optimization, and system-level control for improved battery safety and longevity under realistic operating conditions.

## Data Availability

Not applicable. This manuscript does not report data generation or analysis.

## List of symbols

C_p_: Specific heat capacity($\:J/(kg\cdot K)$)
CV: Coefficient of variation($\:\%$)
t: Time($$\:s$$)
T: Temperature($$\:K$$)
$T_i$: Temperature of cell $\:i$($\:K$)
$${\Delta\:}{T}$$: Temperature difference (T_max_ - T_min_)($$\:K$$)
${\Delta\:}{{T}}_{\mathrm{cell},{i}}$: Intra-cell temperature difference($\:K$)
${\bar T}$: Mean cell temperature($\:K$)
U_0_: Open-circuit voltage($\:V$)
V: Coolant velocity vector($\:m/s$)
V: Cell volume($$\:m^3$$)
$${k}$$: Thermal conductivity($$\:W/m\cdot\:K$$)
${\dot q}$: Volumetric heat generation rate($\:W/{m}^{3}$)
$$p$$: Pressure(Pa)
$$V$$: Cell volume($$\:{m}^{3}$$)
I: Discharge current(A)
$$R_i$$: Internal resistance($$\:\varOmega\:$$)

## Greek symbols

µ: Dynamic viscosity($$\:Pa\cdot s$$)
ρ: Density($$\:kg/m^3$$)
σ: Standard deviation of cell temperature($\:K$)

## Subscripts

cell: Quantity evaluated over an individual cell
f: Fluid (coolant) domain
i: Cell index / internal
max: Maximum value
min: Minimum value
p: At constant pressure (specific heat)
s: Solid domain
t: Temperature-based statistical quantity

## Superscripts and accents

x̄: Overbar – time-mean (average) value, e.g. T̄
ẋ: Overdot – time rate of change, e.g. q̇

## Abbreviations

BMS: Battery Management System
BTMS: Battery Thermal Management System
CAD: Computer-Aided Design
C-DFIC: Channeled Dielectric Fluid Immersion Cooling
CFD: Computational Fluid Dynamics
CV: Coefficient of Variation
EV: Electric Vehicle
FEA: Finite Element Analysis
GV-CNF: Gradually Varied Circular Notched Fins
Li-ion: Lithium-Ion
LTO: Lithium Titanate Oxide
MAE: Mean Absolute Error
NMC: Nickel Manganese Cobalt (oxide)
PCM: Phase Change Material
RMSE: Root Mean Square Error
SIMPLE: Semi-Implicit Method for Pressure-Linked Equations
SOC: State of Charge
SOH: State of Health
TIM: Thermal Interface Material
US06: US06 Aggressive (high-speed) Drive Cycle
